# Gut microbiome dysbiosis in Alzheimer’s disease and mild cognitive impairment: A systematic review and meta-analysis

**DOI:** 10.1371/journal.pone.0285346

**Published:** 2023-05-24

**Authors:** Sherlyn Jemimah, Chahd Maher Musthafa Chabib, Leontios Hadjileontiadis, Aamna AlShehhi

**Affiliations:** 1 Department of Biomedical Engineering, Khalifa University, Abu Dhabi, United Arab Emirates; 2 Healthcare Engineering Innovation Center (HEIC), Khalifa University, Abu Dhabi, United Arab Emirates; 3 Department of Electrical and Computer Engineering, Aristotle University of Thessaloniki, Thessaloniki, Greece; UAE University: United Arab Emirates University, UNITED ARAB EMIRATES

## Abstract

**Background:**

Alzheimer’s disease (AD) is a neurodegenerative disorder that causes gradual memory loss. AD and its prodromal stage of mild cognitive impairment (MCI) are marked by significant gut microbiome perturbations, also known as gut dysbiosis. However, the direction and extent of gut dysbiosis have not been elucidated. Therefore, we performed a meta-analysis and systematic review of 16S gut microbiome studies to gain insights into gut dysbiosis in AD and MCI.

**Methods:**

We searched MEDLINE, Scopus, EMBASE, EBSCO, and Cochrane for AD gut microbiome studies published between Jan 1, 2010 and Mar 31, 2022. This study has two outcomes: primary and secondary. The primary outcomes explored the changes in *α*-diversity and relative abundance of microbial taxa, which were analyzed using a variance-weighted random-effects model. The secondary outcomes focused on qualitatively summarized *β*-diversity ordination and linear discriminant analysis effect sizes. The risk of bias was assessed using a methodology appropriate for the included case-control studies. The geographic cohorts’ heterogeneity was examined using subgroup meta-analyses if sufficient studies reported the outcome. The study protocol has been registered with PROSPERO (CRD42022328141).

**Findings:**

Seventeen studies with 679 AD and MCI patients and 632 controls were identified and analyzed. The cohort is 61.9% female with a mean age of 71.3±6.9 years. The meta-analysis shows an overall decrease in species richness in the AD gut microbiome. However, the phylum *Bacteroides* is consistently higher in US cohorts (standardised mean difference [SMD] 0.75, 95% confidence interval [CI] 0.37 to 1.13, p < 0.01) and lower in Chinese cohorts (SMD -0.79, 95% CI -1.32 to -0.25, p < 0.01). Moreover, the *Phascolarctobacterium* genus is shown to increase significantly, but only during the MCI stage.

**Discussion:**

Notwithstanding possible confounding from polypharmacy, our findings show the relevance of diet and lifestyle in AD pathophysiology. Our study presents evidence for region-specific changes in abundance of *Bacteroides*, a major constituent of the microbiome. Moreover, the increase in *Phascolarctobacterium* and the decrease in *Bacteroides* in MCI subjects shows that gut microbiome dysbiosis is initiated in the prodromal stage. Therefore, studies of the gut microbiome can facilitate early diagnosis and intervention in Alzheimer’s disease and perhaps other neurodegenerative disorders.

## Introduction

Alzheimer’s disease (AD) is a neurodegenerative disorder characterized by a gradual loss of cognition and memory. It is expected that 78 million older adults will be diagnosed with AD by 2030 [1]. AD is preceded by a prodromal or early stage in which patients suffer mild cognitive impairment (MCI) [1]. While there is no known cure for AD, a few studies have reported success in improving cognition with non-drug interventions such as fecal microbial transplants [2, 3] and probiotics in early stages [4].

One possible route of intervention for Alzheimer’s may be the gut microbiome, an ecosystem of about 100 trillion commensal microorganisms representing a distinct group of 500–1000 species [5]. The importance of the gut microbiome in metabolite secretion, pathobiont restriction, and immune system maturation is well-known [5]. The gut microbiome primarily influences neurological function through the gut-brain axis, a channel of communication between the brain and the abdominal organs, through the nervous system and neuromodulator production [6].

Perturbations in gut microbiome composition, termed dysbiosis, have been linked to several diseases [7]. In neurodegenerative disorders, the pathway between gut dysbiosis and neurodegeneration includes immune activation through a defective gut barrier, induction of a systemic inflammatory response, impairment of the blood-brain barrier, and neuroinflammation [7]. Case-control studies of AD identify significant changes in microbial composition, with a greater abundance of pro-inflammatory bacterial genera such as *Escherichia-Shigella*, and a decrease in anti-inflammatory species such as *E. rectale* [8]. However, till date and to the best of our knowledge, no specific microbial taxa have been consistently and uniquely associated with Alzheimer’s. Furthermore, case-control studies with smaller samples may be affected by low power and confounding factors which obscure true biological signals. With several case-control studies being published in recent years, it seems an appropriate next step to pool the studies together in a meta-analysis to derive robust insights with potential for clinical impact.

In this work, we systematically reviewed case-control studies of AD and MCI patient gut microbiomes. The outcomes of gut microbiome studies namely, changes in *α*-diversity, *β*-diversity, changes in relative abundance, and linear discriminant analysis effect sizes, were studied to identify the microbial taxa consistently impacted by gut dysbiosis. Growing evidence relating gut dysbiosis to Alzheimer’s and other forms of dementia emphasizes the timely nature of this article.

## Materials and methods

The study protocol for this systematic review and meta-analysis was finalized in advance of data collection and has been registered with The International Prospective Register of Systematic Reviews (PROSPERO), number CRD42022328141 (accessible at https://www.crd.york.ac.uk/PROSPERO/display_record.php?RecordID=328141). The protocol was written according to PRISMA (Preferred Reporting Items for Systematic reviews and Meta-Analyses) guidelines. A PRISMA Protocols (PRISMA-P) checklist of items addressed in the systematic review protocol can be found in S1 Table.

### Search strategy

We searched MEDLINE, Cochrane, EBSCO, EMBASE, and Scopus, for case-control metagenomic and 16S studies of Alzheimer’s disease (AD) and mild cognitive impairment (MCI) in humans. The search was limited to papers written in English and published between Jan 1, 2010, and Mar 31, 2022. A set of controlled vocabulary terms related to Alzheimer’s and metagenomics were formulated and combined with the ‘AND’ operator to generate search queries. The controlled vocabulary has been provided in S2 Table.

### Inclusion and exclusion criteria

Studies were eligible if they assessed the gut microbiomes of human patients with AD or MCI with metagenomic sequencing and reported outcomes such as *α*-diversity, *β*-diversity ordination, relative abundances of various taxa, and linear discriminant analysis effect sizes (LEfSe). Eligible study designs were case-control and intervention studies (with baseline sampling). Patients had to meet well-defined diagnostic criteria such as DSM (Diagnostic and Statistical Manual of Mental Disorders) or NIA-AA (National Institute on Aging and Alzheimer’s Association) guidelines. If patients self-reported decreases in cognition and memory (corroborated by a caregiver) without meeting the diagnostic criteria for AD, they were considered to have MCI. Controls were cognitively normal (CN) subjects, reasonably matched to the AD and MCI cohorts in age, gender, years of education, and lifestyle. Exclusion criteria included antibiotic use within two weeks of sample collection and the presence of confounding conditions such as depression, cancer, or any other genetic/neurological disorders.

### Study selection and data extraction

All identified records were imported into Rayyan, a widely-used mobile and web application that helps expedite the initial screening of records for systematic review [9], for de-duplication and screening. De-duplication was automatic if records were matched 100% and manually performed if the similarity ranged from 80–99%. Titles and abstracts were initially screened for eligibility by two authors. Full-text reports of selected studies were further assessed using the selection criteria by two authors, with a plan to resolve any disagreements by the corresponding author. We also performed manual searches of the reference lists of the included studies. Reported data in tabular and graphical form was extracted, cleaned, and tabulated from the full-text reports. The corresponding authors of included studies were contacted to request any missing data.

### Outcome measures

The primary outcomes of interest were measures of microbial diversity, namely changes in *α*-diversity and relative abundance of various taxa. Microbial diversity refers to the variety (richness) and abundance (evenness) of species in a defined unit of study [10, 11]. In this study, *α*-diversity outcomes such as Shannon, Simpson, Chao index, Abundance-based Coverage Estimators (ACE), and the number of species observed (S_*obs*_) were included. The relative abundances (proportion of a given taxon) at the phylum, family, and genus levels were also examined.

Secondary outcomes of interest included *β*-diversity ordination and LEfSe results. The results of ordination (summarization of distance matrix and projection in a low-dimension space) and statistical testing of *β*-diversity indices (Bray-Curtis, Jaccard, weighted/unweighted UniFrac, Aitchison distances) were examined. The definitions and interpretation of the diversity indices have been provided in S1 Appendix. Additionally, we included a qualitative analysis of LEfSe (linear discriminant analysis effect size) [12]. LEfSe identifies differentially abundant taxa between two groups of metagenomic samples using non-parametric statistical tests and linear discriminant analysis with strict cut-offs.

Where necessary, units were converted so that related outcomes were on consistent scales (for instance, percentage composition was converted to proportion).

### Additional data items

In addition to reported primary and secondary outcomes, the following data were recorded: study location, cohort size, the average age of the cohort, the proportion of female participants, diagnostic and exclusion criteria used for patients, sequencing platform, bioinformatics methods for data analysis, and details of ethical committee/review board approvals.

### Statistical analysis

All statistical analyses were performed using the R programming language (version 4.0.3, The R Foundation for Statistical Computing, Vienna, Austria). The R metaphor package was used for the quantitative synthesis of *α*-diversity and relative abundance data. LEfSe results and *β*-diversity outcomes were summarized qualitatively. For quantitative synthesis, continuous outcomes were reported using the standard mean difference (SMD). Three or more studies measuring the same outcome were combined with an inverse-variance weighted random-effects model. Reported median and interquartile range values were converted to estimates of the mean and standard deviation (SD) [13]. Means with 95% CIs were converted to means and SDs using the formula provided by the Cochrane Handbook. Forest plots were generated for each primary outcome of interest, with the weight indicating the influence of an individual study on the pooled result. Heterogeneity was quantified using *τ*^2^ (tau^2^) and *I*^2^ statistics. We examined the significance of heterogeneity using the *χ*^2^ (chi^2^) test. A P value less than 0.05 was considered statistically significant. If significant heterogeneity was present, we performed a subgroup meta-analysis by grouping studies according to sample characteristics, provided that sufficient studies reported the outcome. *A priori* variables identified for the subgroup analysis were age, sex and study location.

### Risk of bias and quality assessment

Funnel plots were assessed for outcomes with seven or more studies (i.e., the Shannon index and the relative abundances of phylum *Bacteroidetes* in AD patients) to detect publication bias. The risk of bias was assessed using criteria appropriate for the selected study designs [14]. We did not formally assess the quality of the included studies, as the outcome data was generated from next-generation sequencing methods and analyzed using standard bioinformatics workflows such as QIIME.

## Results

**Fig 1:**
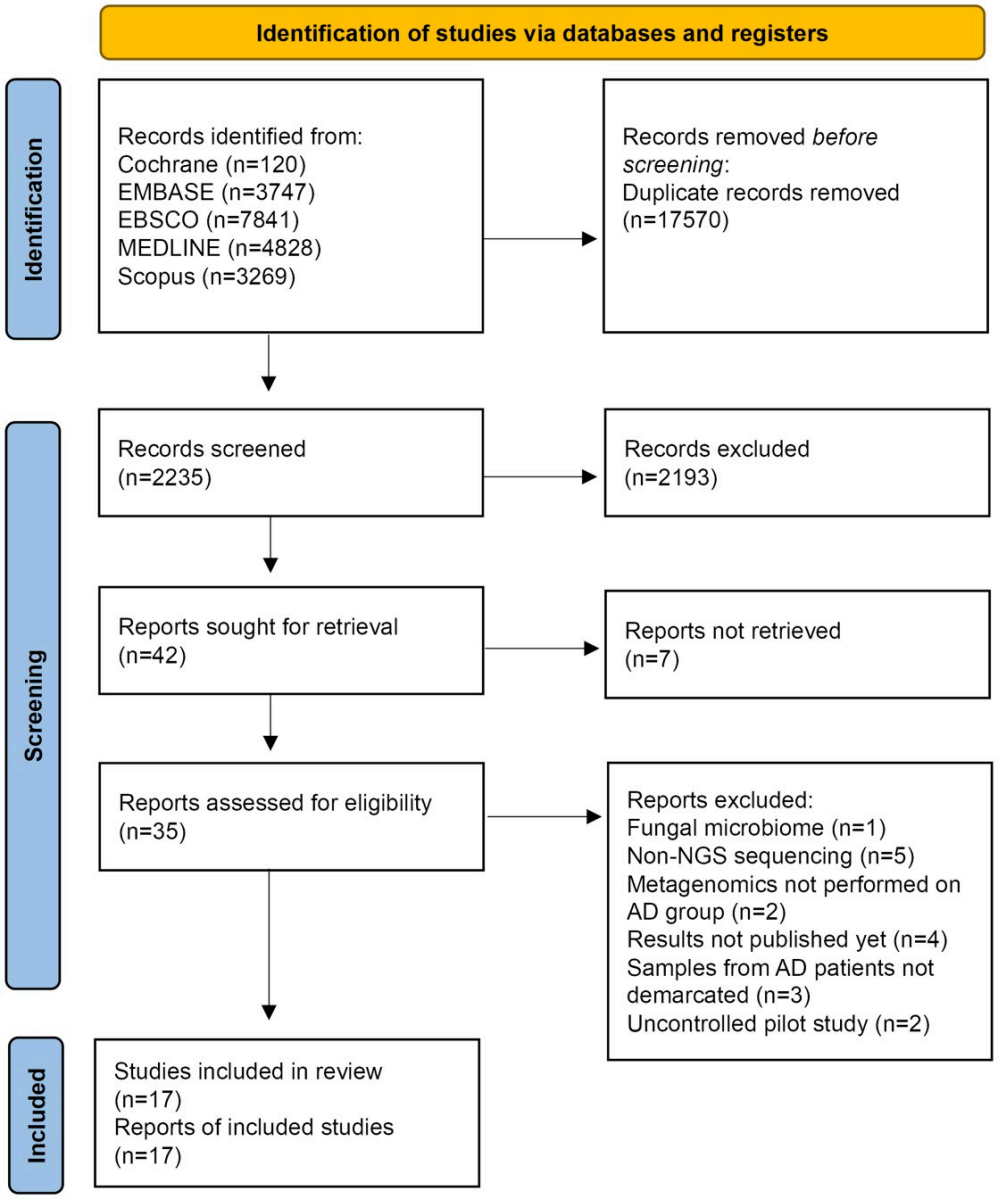
Study selection. PRISMA flow diagram of selected studies for inclusion.

Of 2235 records identified, 42 full-text articles were screened, of which 17 publications of 17 unique studies were deemed eligible and included in the narrative synthesis (S3 Table). The study selection process has been summarized in Fig 1. All included studies were deemed to provide sufficient data for inclusion for our study. An overview of the characteristics of each study is in S4 Table. All 17 included studies identify significant alterations in gut microbiome composition of AD or MCI patients compared to CN subjects.

The 17 included studies comprise 679 patients (241 with MCI and 438 with AD), and 632 controls. The cohorts were 61.9% female with a mean age of 71.3±6.9 years. The cohorts in the included studies ranged in size from 11 to 46 MCI patients and 7 to 100 AD patients. The corresponding controls (cognitively normal or CN group) were matched with the MCI and AD patients in terms of age and proportion of female participants. The controls also led a lifestyle similar to the MCI and AD patients. All studies have used well-defined diagnostic criteria such as DSM and NIA-AA guidelines for AD/MCI patient inclusion. Twelve studies analyzed AD cohorts and ten studies analyzed MCI cohorts. Most studies were undertaken in China (n = 11; 65%). Two studies were randomized controlled trials [15, 16], and two were derived from longitudinal studies [17, 18]. The four studies reported primary outcomes at baseline and were therefore included in the study. The remaining were case-control studies. Most included studies have reported co-morbidities in their cohorts. Twelve studies reported the proportion of participants with diabetes (3.1 to 33.3% for CN, 9.4 to 13.6% for MCI, and 6.7 to 23.3% for AD) [17, 19–29] and eight studies reported the proportion of participants with hypertension (19.2 to 75% for CN, 9.1 to 72.2% for MCI and 11.0 to 43.0% for AD) [19–21, 23–26, 28, 29]. Five studies reported the proportion of participants with cardiovascular disease (CVD; 11.3 to 15.6% for CN subjects, 22.7% for MCI, and 8.3 to 18.6% for AD) [19, 21, 23, 26, 28, 29] Five studies [15, 16, 18, 30, 31] did not report co-morbidities in their cohorts.

Most included studies used the Illumina MiSeq platform to sequence V3-V4 regions of bacterial 16S rRNA. Exceptions include Pan et al. [26] who sequenced the V1-V9 region, and Nagpal et al. [16] and Vogt et al. [27], who sequenced the V4 region. Haran et al. [17] performed shotgun sequencing with Illumina NextSeq 500, and therefore, their data were included in the synthesis of *β*-diversity outcomes. Although there were some heterogeneity in the bioinformatic analyses, the introduction of QIIME (Quantitative Insights Into Microbial Ecology) software [32] has provided scientists with a uniform framework for the analysis of 16S data. The majority of the included studies have employed QIIME. The bioinformatics methods and taxonomic units used for each included study in have been summarized in S5 Table.

### *α*-diversity

**Fig 2:**
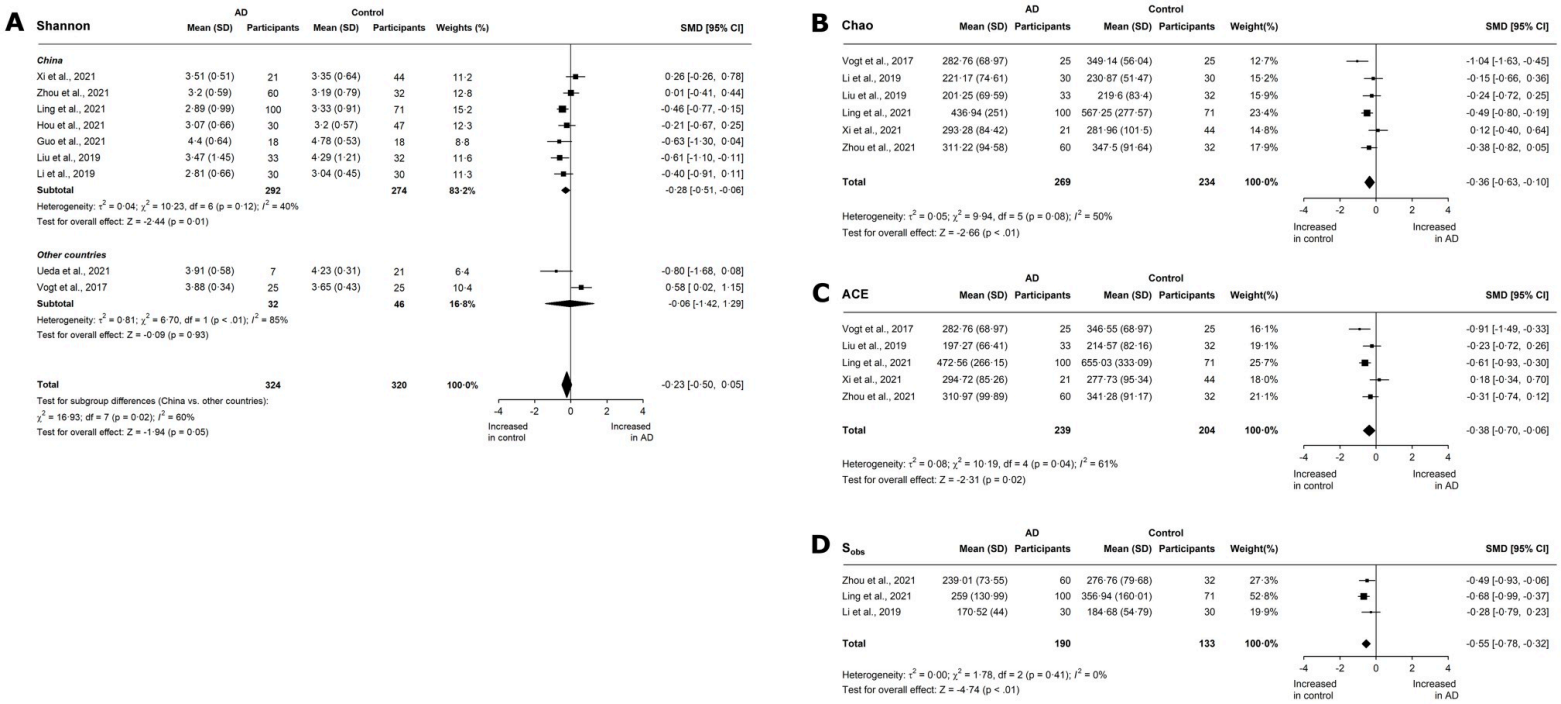
Meta-analysis of *α*-diversity outcomes in AD cohorts. Forest plots for (A) Shannon index with subanalysis by location, (B) Chao, (C) ACE, and (D) S_obs_ in AD cohorts. Data are mean (SD) and standard mean difference (95% CI) between groups by random-effect meta-analysis.

**Fig 3:**
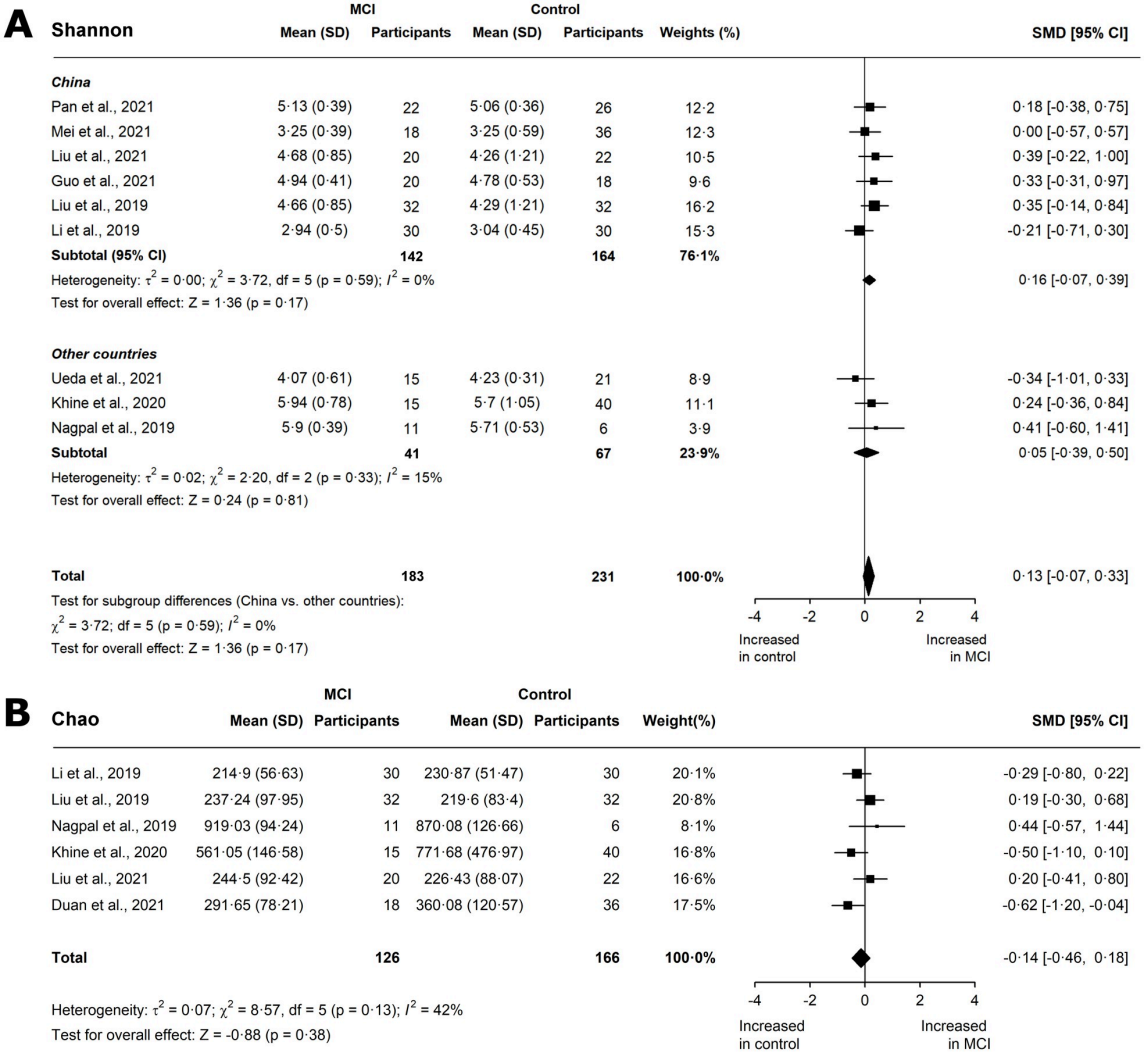
Meta-analysis of *α*-diversity outcomes in MCI cohorts. Forest plots for (A) Shannon index with subanalysis by location, and (B) Chao in MCI cohorts. Data are mean (SD) and standard mean difference (95% CI) between groups by random-effect meta-analysis.

Shannon and Simpson’s indices are indicative of both richness and evenness. Nine studies reported the Shannon index for AD patients [18, 20–24, 27, 28, 30]. Overall, a small, significant decrease was observed in Shannon diversity (SMD -0.23, 95% CI -0.5 to 0.05; p = 0.05; Fig 2A) with substantial, significant heterogeneity (I^2^ = 60%; p < 0.01). A subgroup meta-analysis by location showed a small, significant reduction in Chinese cohorts (SMD -0.28, 95% CI -0.51 to -0.06; p = 0.01), with low heterogeneity (I^2^ = 40%; p = 0.12). We also observed a small, insignificant increase in the Shannon diversity of MCI patients, irrespective of location (SMD 0.13, 95% CI -0.07 to 0.33; p = 0.17, Fig 3A). The meta-analysis of the Simpson index is provided in the Extended Results S2 Appendix.

In terms of species richness, three studies report the number of species observed (S_obs_) for AD patients [22, 23, 28]. There was a significant, moderate reduction in the number of species (SMD -0.55, 95% CI -0.78 to -0.32; p < 0.01, Fig 2D), and no heterogeneity (I^2^ = 0%; p = 0.41). In five studies that reported ACE in AD [22, 23, 27, 28, 30], a significant, moderate reduction was found (SMD -0.38, 95% CI -0.7 to -0.06; p = 0.02, Fig 2C), although heterogeneity was high (I^2^ = 61.9%; p = 0.04). An insufficient number of studies reported S_obs_ and ACE for MCI patients. Six studies reported Chao indices for AD patients [22–24, 27, 28, 30]. A moderate, significant decrease was observed (SMD -0.36, 95% CI -0.63 to -0.10; p < 0.01; Fig 2B), with low heterogeneity across the studies (I^2^ = 48.3%; p = 0.08). Furthermore, a small, insignificant decrease was observed in MCI patients (SMD -0.14, 95% CI -0.46 to 0.18; p = 0.38, Fig 3B), with low heterogeneity (I^2^ = 42%; p = 0.13).

### *β*-diversity ordination

Most studies have reported the results of ordination and statistical tests of *β*-diversity indices. Commonly-used techniques for ordination include Principal Component Analysis (PCA) [16, 19, 20, 22, 24], Non-metric Multidimensional Scaling (NMDS) [29, 34], and Principal Co-ordinates Analysis (PCoA) [15, 18, 19, 21, 23, 26, 28, 30, 31]. Differences between groups have been tested using PERMANOVA (PERmutational Multivariate ANalysis Of VAriance) [15, 17, 18, 23, 24, 26–28, 30, 31] and ANOSIM (ANalysis Of SIMilarity) [19, 21, 22]. The magnitude and significance of differences in *β*-diversity among AD, MCI and CN have been summarized in Table 1, and a qualitative summary is provided in the Extended Results S2 Appendix.

**Table 1:** Summary of findings from analysis of *β*-diversity outcomes.

Study	Comparison	*β*-diversity	Ordination	Statistical testing	Findings
Duan et al., 2021	CN/AD/MCI	Bray-Curtis	PCA, PCoA	PCA ANOSIM R^2^ = 0.0375 (p = 0.026); PCoA ANOSIM R^2^ = 0.0416 (p = 0.004)	Species diversity was clearly different among the three groups
Guo et al., 2021	AD vs CN	Bray-Curtis		Wilcoxon rank-sum test (p = 0.016)	Significant difference between AD and CN
	MCI vs CN	Bray-Curtis		Wilcoxon rank-sum test (p = 0.017)	Significant difference between MCI and CN
	AD vs CN	Weighted UniFrac		Wilcoxon rank-sum test (p < 0.001)	Significant difference between AD and CN
	MCI vs CN	Weighted UniFrac		Wilcoxon rank-sum test (p < 0.001)	Significant difference between MCI and CN
Haran et al., 2019	AD vs CN	Jaccard	t-SNE^a^	PERMANOVA (p = 0.001)	Elders with AD cluster away from those without dementia
Hou et al., 2021	AD vs CN	Bray-Curtis	PCoA	ANOSIM R^2^ = 0.028 (p = 0.039)	Slight difference in gut microbial composition between groups
	AD vs CN	Weighted UniFrac	PCoA	ANOSIM R^2^ = 0.025 (p = 0.065)	No distinguishable bacterial microbiota between AD and CN
	AD vs CN	Unweighted UniFrac	PCoA	ANOSIM R^2^ = 0.023 (p = 0.233)	No distinguishable bacterial microbiota between AD and CN
Khine et al., 2020	MCI vs CN	Weighted and unweighted UniFrac	PCoA	PERMANOVA R^2^ = 0.07 (p = 0.0012)	Significant differences between the CN and MCI groups
Li et al., 2019	CN/AD/MCI	Weighted UniFrac	PCA	ANOSIM R = 0.345 (p = 0.001)	Significant differences among the three groups; however, the differences between AD and MCI were not significant
	CN/AD/MCI	Unweighted UniFrac		ANOSIM R = 0.22 (p = 0.001)	
Ling et al., 2021	AD vs CN	Jaccard, Bray-Curtis, weighted and unweighted UniFrac	PCoA	PERMANOVA (p < 0.01)	Divided the AD and CN groups into different clusters
Liu et al., 2019	AD vs CN	Bray-Curtis, weighted and unweighted UniFrac	PCA	PERMANOVA for Bray-Curtis R = 0.182 (p = 0.017); weighted UniFrac R = 0.184 (p = 0.067); unweighted UniFrac R = 0.134 (p = 0.183)	Significant compositional differences between AD and CN groups in terms of Bray-Curtis but not weighted or unweighted UniFrac
	AD vs MCI	Bray-Curtis, weighted and unweighted UniFrac	PCA	PERMANOVA for Bray-Curtis R = 0.197 (p = 0.005); weighted UniFrac R = 0.274 (p = 0.003); unweighted UniFrac R = 0.148 (p = 0.044)	Significant compositional differences between AD and MCI groups
	MCI vs CN	Bray-Curtis, weighted UniFrac, and unweighted UniFrac	PCA	PERMANOVA for Bray-Curtis R = 0.176 (p = 0.012); weighted UniFrac R = 0.2226 (p = 0.01); unweighted UniFrac R = 0.138 (p = 0.138)	Significant compositional differences between MCI and CN groups in terms of Bray-Curtis and the weighted but not unweighted UniFrac
Nagpal et al., 2019	MCI vs CN	Weighted UniFrac	PCA		No notable difference in terms of *β*-diversity
Pan et al., 2021	MCI vs CN	Weighted UniFrac	PCoA, NMDS	PERMANOVA (p = 0.048)	Gut microbiota profiles of the MCI cases clustered apart from those of control subjects
Ueda et al., 2021	CN/AD/MCI	Bray-Curtis	PCoA	PERMANOVA between MCI and CN groups, R^2^ = 0.0465 (p = 0.0968); between AD and CN groups R^2^ = 0.0534 (p = 0.1423)	Genus composition does not differ significantly among the three groups
Vogt et al., 2017	AD vs CN	Bray-Curtis, weighted UniFrac and unweighted UniFrac	NMDS	PERMANOVA for Bray-Curtis distance F = 2.87 (p < 0.001); weighted UniFrac F = 3.84 (p < 0.001); unweighted UniFrac F = 2.60 (p < 0.005)	Demonstrated compositional differences in the microbiome between AD and CN groups
Xi et al., 2021	AD vs CN	Bray-Curtis	PCoA	PERMANOVA R^2^ = 0.025 (p = 0.027)	Significant differences between AD and CN
Yıldırım et al., 2022	CN/AD/MCI	Bray-Curtis and Jaccard	PCoA	PERMANOVA (p = 0.0001)	Highly significant separation of the three groups
	CN/AD/MCI	Aitchison	PCA	PERMANOVA (p = 0.02)	The three groups are distinct
Zhou et al., 2021	AD vs CN	Weighted and unweighted UniFrac	PCoA	PERMANOVA of unweighted UniFrac: R^2^ = 0.029 (p < 0.001); weighted UniFrac: R^2^ = 0.026 (p = 0.026)	Significant differences between the AD group and the CN group
Zhuang et al., 2018	AD vs CN	Weighted UniFrac	PLS-DA^b^		Weighted UniFrac distance matrix was used to successfully cluster samples by family, genus, species and OTU levels.

^a^ t-stochastic neighbor embedding;

^b^ partial least squares discriminant analysis

### Relative abundance

**Fig 4:**
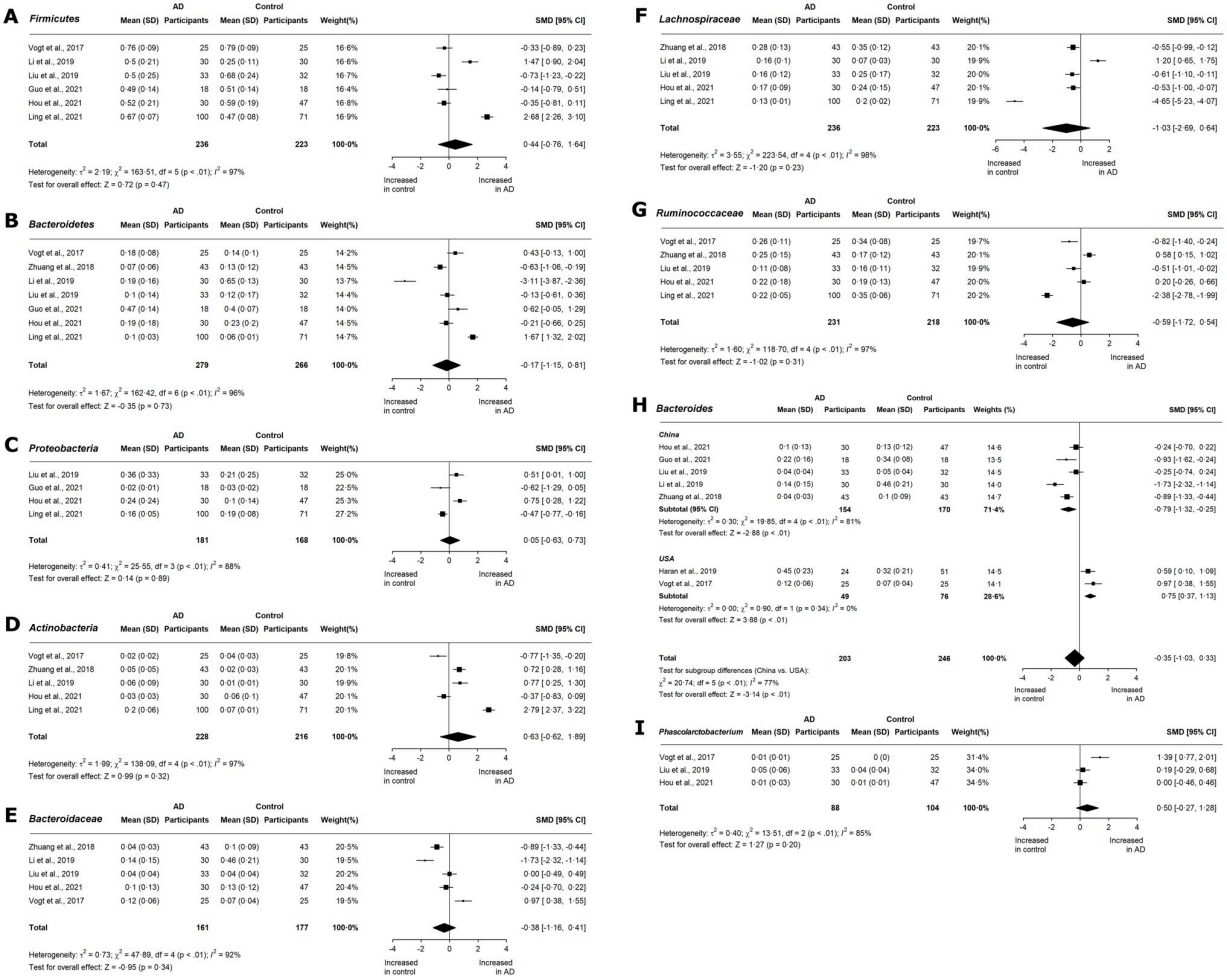
Meta-analysis of reported relative abundances in AD cohorts. Forest plots for relative abundance of phyla A) *Firmicutes*, B) *Bacteroidetes*, C) *Proteobacteria*, and D) *Actinobacteria*; families E) *Bacteroidaceae*, F) *Lachnospiraceae*, and G) *Ruminococcaceae*; and genera H) *Bacteroides*, and I) *Phascolarctobacterium* in AD cohorts. Data are mean (SD) and standard mean difference (95% CI) by random-effect meta-analysis.

**Fig 5:**
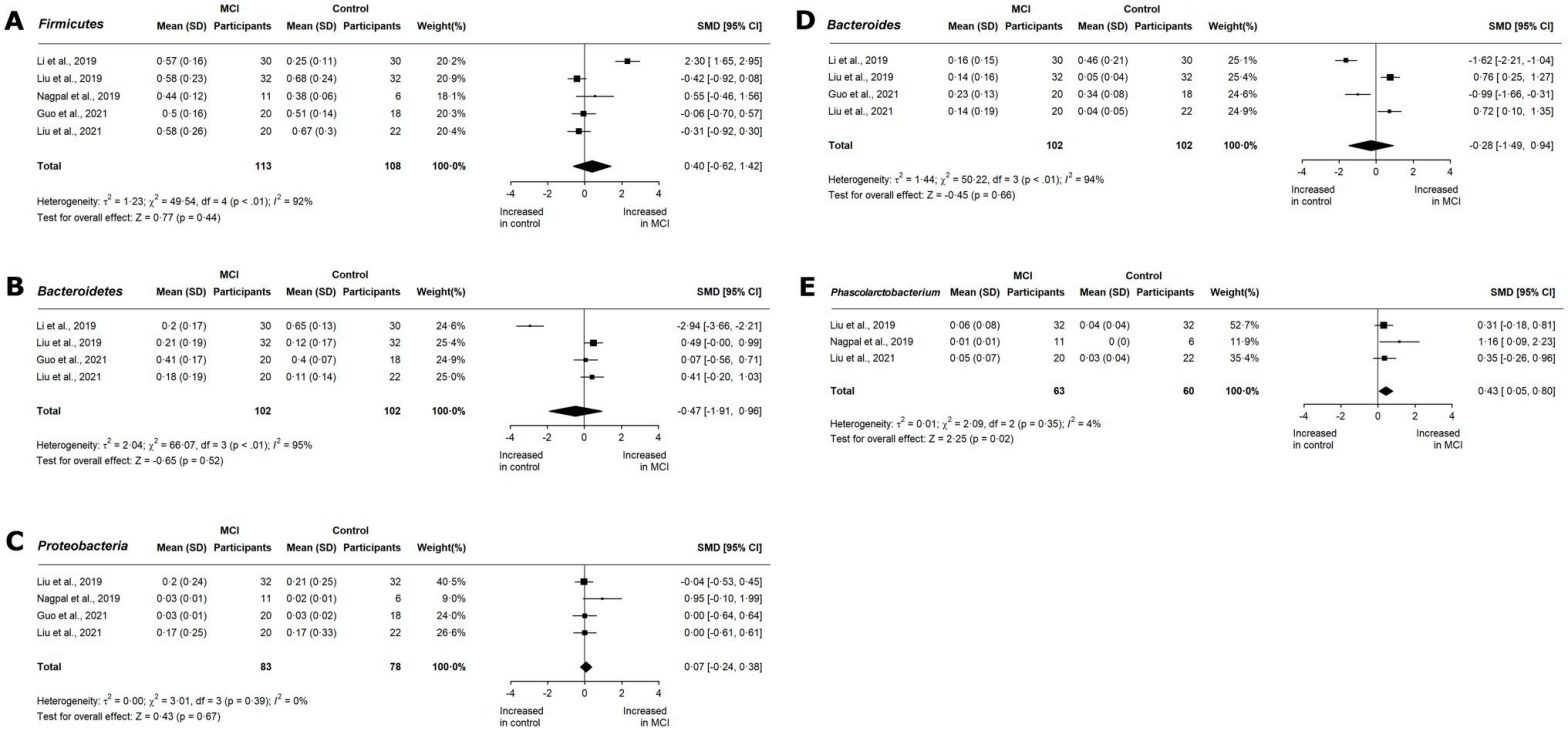
Meta-analysis of reported relative abundances in MCI cohorts. Forest plots for relative abundance of phyla (A) *Firmicutes*, (B) *Bacteroidetes*, and (C) *Proteobacteria*; and genera (D) *Bacteroides*, and (E) *Phascolarctobacterium* in MCI cohorts. Data are mean (SD) and standard mean difference (95% CI) by random-effect meta-analysis.

In terms of relative abundance, *Bacteroidetes* and *Firmicutes* are the dominant phyla and comprise 90% of the gut microbiome [33]. *Firmicutes* encompasses Gram-positive anaerobes and aerobes [34]. Eight studies have reported the relative abundance of *Firmicutes* in AD [20–24, 27] and MCI [16, 20, 22, 24, 25]. In both AD (SMD 0.44, 95% CI -0.76 to 1.64; p = 0.47; Fig 4A) and MCI (SMD 0.40, 95% CI -0.62 to 1.42; p = 0.44; Fig 5A), the meta-analyses were marked by considerable heterogeneity. The *Bacteroidetes* phylum includes Gram-negative anaerobes and aerobes [34], and is composed primarily of *Bacteroides* and *Prevotella* genera. *Bacteroidetes* phylum was shown to decrease slightly in AD (SMD -0.17, 95% CI -1.15 to 0.81; p = 0.73; Fig 4B), with substantial, significant heterogeneity (I^2^ = 96%; p < 0.01). Four studies reported changes in the relative abundance of *Bacteroidetes* in MCI participants [20, 22, 24, 25]. From the forest plot, a moderate, insignificant decrease was seen (SMD -0.47, 95% CI -1.91 to 0.96; p = 0.52; Fig 5B) with significant heterogeneity (I^2^ = 95%; p < 0.01).

The *Actinobacteria* and *Proteobacteria* phyla represent 10% of the gut microbiome. The aging gut is marked by an increase in *Proteobacteria* [35], a highly heterogenous taxon of facultative anaerobes. Four studies have reported the relative abundance of *Proteobacteria* in AD and MCI patients. In AD patients, the standardized mean differences of individual studies ranged from -0.62 to 0.75. Overall, no significant effect was observed (SMD 0.05, CI -0.63 to 0.73; p = 0.89; Fig 4C), but there was significant heterogeneity (I^2^ = 88%; p < 0.01). In MCI patients, there was no change in the abundance of Proteobacteria (SMD 0.07, CI -0.24 to 0.38; p = 0.67; Fig 5C). In contrast, the *Actinobacteria* phylum showed higher relative abundance in two AD cohorts. *Actinobacteria* are mainly represented by *Bifidobacteriaceae*, and may have antidepressant effects through tryptophan production [36]. Elderly individuals have lower levels of *Actinobacteria* [35]. Overall, there was a moderate but insignificant increase in AD (SMD 0.63, 95% CI -0.62 to 1.89; p = 0.32; Fig 4D), with substantial, significant heterogeneity (I^2^ = 97%; p < 0.01). While Li et al. (2019) [22] reported a moderate increase in *Actinobacteria* in MCI patients, no other studies have reported *Actinobacteria* abundance at the MCI stage.

Five studies have reported the relative abundances of the family *Lachnospiraceae* in AD patients [21–24, 29] which along with *Ruminococcaceae*, is associated with non-specific shifts in disease and the production of small-chain fatty acids (SCFAs) [37]. There was a large insignificant decrease in AD (SMD -1.03, 95% CI -2.69 to 0.64; p = 0.23; Fig 4F), with substantial, significant heterogeneity (I^2^ = 98%; p < 0.01). A similar trend was observed in the five studies reporting *Ruminococcaceae* abundance [21, 23, 24, 27, 29], with a moderate, insignificant decrease (SMD -0.59, 95% CI -1.72 to 0.54; p = 0.31; Fig 4G) and substantial heterogeneity (I^2^ = 97%; p < 0.01). An insufficient number of studies have reported the relative abundance of *Lachnospiraceae* and *Ruminococcaceae* for MCI.

The *Bacteroidaceae* family consists of obligate anaerobic Gram-negative bacteria, and reduces in abundance with age [38]. There was a small reduction in *Bacteroidaceae* abundance in AD (SMD -0.38, 95% CI -1.16 to 0.41; p = 0.34; Fig 4E) with substantial heterogeneity (I^2^ = 92%; p < 0.01). This reduction is a feature of Chinese cohorts, and is in contrast to the meta-analysis of metagenomic studies in Parkinson’s disease, which revealed no significant differences [39].

*Bacteroides* utilize polysaccharides to produce acetate and propionate [40, 41] Our study showed a significant, moderate reduction in *Bacteroides* in AD (SMD -0.35, 95% CI -1.03 to 0.33; p < 0.01; Fig 4H). A subgroup meta-analysis by location revealed decreased abundance in Chinese cohorts (SMD -0.79, 95% CI -1.32 to -0.25; p < 0.01) and increased abundance in US cohorts (SMD 0.75, 95% CI 0.37 to 1.13; p < 0.01). All studies reporting *Bacteroides* abundance in MCI were conducted in China, and showed an insignificant reduction (SMD -0.28, 95% CI -1.49 to 0.94; p = 0.66; Fig 5D) with high heterogeneity (I^2^ = 94%; p < 0.01).

*Phascolarctobacterium* utilize succinate to produce propionate and acetate [42]. The *Phascolarctobacterium* genus was moderately increased in AD (SMD 0.50, 95% CI -0.27 to 1.28; p = 0.20; Fig 4I) with substantial heterogeneity. In MCI, *Phascolarctobacterium* showed a significant, moderate increase (SMD 0.43, 95% CI 0.05 to 0.80; p = 0.02; Fig 5E) with low heterogeneity (I^2^ = 4%; p = 0.35).

### Linear discriminant analysis effect sizes (LEfSe)

All nine studies which reported LEfSe to identify differentially abundant taxa between AD/MCI and CN participants [20–24, 26, 28–30] have been included for the following qualitative summary. Key similarities and differences in the commonly-represented taxa, i.e., phyla, families, and genera, have been compiled in Table 2. LEfSe analysis uses statistical testing and modeling with a rigorous cut-off to determine the most discriminative features between the two groups. Moreover, the false positive rate was reported to be lower than 0.05. Therefore, a taxon was deemed consistent if it was reported by two or more studies to be of higher abundance (or lower abundance) in AD compared to controls, and there were no studies which report contrasting LEfSe scores for the same taxon. We have included information on the score cut-offs to reflect the strictness of the comparison.

**Table 2:** Summary of LEfSe calculations of relative abundances.

Taxon	Zhuang 2018	Li 2019	Liu 2019	Guo 2021	Zhou 2021	Xi 2021	Hou 2021	Ling 2021	Pan 2021
LDA score cut-off	2	2	2	2	2	2.5	3	3	NA^a^
*Actinobacteria*	↑^b^							↑	
*Bacteroidetes*									↓
*Firmicutes*								↓	
*Proteobacteria*			↑	↓			↑		
*Bacteroidaceae*	↓			↓					↓
*Bifidobacteriaceae*					↑				
*Clostridiaceae*			↑						
*Clostridiaceae 1*								↓	
*Enterobacteriaceae*			↑				↑		
*Enterococcaceae*							↓	↑	
*Eubacteriaceae*					↓			↑	
*Lactobacillaceae*					↑			↑	
*Lachnospiraceae*	↓			↓				↓	
*Prevotellaceae*				↑				↓	↓
*Ruminococcaceae*	↑								
*Akkermansia*		↑							
*Alistipes*		↓							
*Alloprevotella*		↓				↑			↓
*Anaerostipes*							↓	↓	
*Bacteroides*	↓	↓		↓					↓
*Bifidobacterium*		↑			↑			↑	
*Blautia*		↑		↓	↑				
*Clostridium IV*								↑	
*Clostridium VIII*								↑	
*Clostridium XIVa*					↑			↑	
*Clostridium sensu stricto*								↓	
*Dorea*		↑		↓					
*Eubacterium*					↓			↑	
*Faecalibacterium*						↑		↓	
*Lachnospira*				↓					↓
*Lactobacillus*		↑			↑			↑	↑
*Paraprevotella*		↓		↑					
*Phascolarctobacterium*				↓					↑
*Prevotella*	↑	↓						↓	

^a^ Not available; Up arrow (↑) indicates the relative abundance of the taxa is higher in AD patients’ microbiota. The down arrow (↓) indicates lower relative abundance in AD patients’ microbiota compared to cognitively normal controls.

Six studies reported LEfSe for *Actinobacteria*, *Bacteroidetes*, *Firmicutes* and *Proteobacteria*. Two studies reported an increased abundance of *Actinobacteria* in AD [23, 29] In the case of *Proteobacteria*, two studies [21, 24] reported an increased abundance while one study reported decreased abundance [20]. Reduced abundance was also observed for *Firmicutes* [23] and *Bacteroidetes* [26]. *Bacteroidaceae* [20, 26, 29] and *Lachnospiraceae* [20, 23, 29] were consistently lower, whereas *Enterobacteriaceae* [21, 24] and *Lactobacillaceae* [23, 28] were consistently higher in abundance. *Bifidobacteriaceae* [28] and *Ruminococcaceae* [29] also showed higher abundance. At the genus level, four studies reported consistent reduction in *Bacteroides* [20, 22, 26, 29]. Other genera with consistently lower abundance included *Anaerostipes* [21, 23] and *Lachnospira* [20, 26]. In contrast, *Clostridium* [23, 28], *Lactobacillus* [22, 23, 26, 28] and *Bifidiobacterium* [22, 23, 28] were increased in AD. Additional results have been presented in the Extended Results S2 Appendix.

### Risk of bias assessment

The overall risk of bias assessment for all included papers has been summarized in Table 3. Four studies showed an unclear risk of bias. One study included selected patients slated to undergo orthopedic surgery [19]. In another study, the elders were part of a longitudinal observation study and thus were not adequately matched [18]. Likewise, in the study by Haran et al. [17], participants were drawn from a longitudinal study of elders in care facilities. We were able to assess selective reporting bias for Shannon index and relative abundance of genus *Bacteroides*. The reporting bias was low according to the funnel plots from the two outcomes, provided in S1 Fig. We deemed the evidence related to primary outcomes of high quality due to the next-generation sequencing methods and established analysis pipelines used to generate the data.

**Table 3:** Summary risk-of-bias assessment for included studies.

Study	Study design and objectives	Selection of participants and constitution of study groups	Other information bias	Statistical methods to control confounding	Statistical methods excluding methods to control confounding	Conflict of interest	Summary risk-of-bias assessment
Duan et al. 2021	Low	Unclear	Unclear	Unclear	Low	Low	Unclear
Guo et al. 2021	Low	Low	Unclear	Low	Low	Low	Low
Haran et al. 2019	Low	Unclear	Unclear	Unclear	Low	Low	Unclear
Hou et al. 2021	Low	Low	Unclear	Unclear	Low	Low	Low
Khine et al. 2020	Low	Low	Unclear	Low	Low	Low	Low
Li et al. 2019	Low	Low	Unclear	Low	Low	Low	Low
Ling et al. 2021	Low	Low	Unclear	Unclear	Low	Low	Low
Liu et al. 2019	Low	Low	Unclear	Unclear	Low	Low	Low
Liu et al. 2021	Low	Low	Unclear	Unclear	Low	Low	Low
Nagpal et al. 2019	Low	Unclear	Unclear	Low	Low	Low	Unclear
Pan et al. 2021	Low	Low	Unclear	Unclear	Low	Low	Low
Ueda et al. 2021	Low	Unclear	Unclear	Unclear	Low	Low	Unclear
Vogt et al. 2017	Low	Low	Unclear	Unclear	Low	Low	Low
Xi et al. 2021	Low	Low	Unclear	Unclear	Low	Low	Low
Yıldırım et al. 2022	Low	Low	Unclear	Unclear	Low	Low	Low
Zhou et al. 2021	Low	Low	Unclear	Unclear	Low	Low	Low
Zhuang et al. 2018	Low	Low	Unclear	Unclear	Low	Low	Low

## Discussion

In this work, gut microbiome studies were systematically reviewed in AD and MCI to obtain insights into the direction and extent of gut dysbiosis in terms of diversity and relative abundances of various taxa. In terms of *α*-diversity, a slight increase was observed in MCI patients. However, in AD patients, there was a slight, significant decrease in the Shannon and Simpson indices. The latter observation is inconsistent with previous meta-analyses of *α*-diversity indices in Parkinson’s disease [43]. Moreover, in AD patients, there were significant decreases in S_obs_, ACE, and the Chao index. MCI patients also showed a small, but insignificant, decrease in Chao index. Better consensus was seen in terms of *β*-diversity, with most studies reporting significant differences among AD, MCI and CN cohorts. Therefore, our meta-analyses of both *α*- and *β*-diversity outcomes suggest significant differences in terms of species richness and less change in evenness.

From the meta-analyses of relative abundances, there were small to moderate, mostly insignificant changes in MCI patients. Although insignificant, the *Firmicutes* phylum increased moderately and the *Bacteroidetes* phylum decreased moderately. The *Bacteroides* genus also shows a small, insignificant decrease. Interestingly, there was a moderate, significant increase in *Phascolarctobacterium*, which was also observed in AD patients but without significance. Moreover, in AD patients, there was a mild, insignificant increase in *Firmicutes* and *Actinobacteria*, a small, insignificant decrease in *Bacteroidetes*, and no change in *Proteobacteria*. At the family level, *Lachnospiraceae* and *Bacteroidaceae* decreased and *Bifidobacteriaceae* increased in abundance, further supported by our analysis of LEfSe. Notably, regional differences were observed in the abundance of the *Bacteroides* genus. Our meta-analysis revealed that *Bacteroides* was reduced in Chinese cohorts but showed an increase in US-based AD cohorts, thus supporting the notion that region, diet and lifestyle may have a considerable influence on the gut composition and therefore, AD pathophysiology. The LEfSe synthesis further corroborates our observation that *Bacteroides* was reduced in Chinese AD cohorts. Additionally, LEfSe results show an increase in *Enterobacteriaceae*, *Lactobacillaceae*, *Bifidobacterium*, *Akkermansia*, and *Clostridium*, and a decrease in *Anaerostipes* and *Lachnospira*.

The gut microbiota is responsible for the production of several metabolites, of which SCFAs are of particular interest for their role in gut health and inflammation. For instance, butyrate has been shown to reduce inflammation and regulate the host immune system [35]. Treatment with sodium butyrate in germ-free mice resulted in decreased blood-brain barrier (BBB) permeability [44]. However, a recent study also linked SCFA supplementation to increased amyloid-*β* burden [45]. Furthermore, the LEfSe synthesis has shown an increased abundance of acetate, lactate, and propionate producers such as *Akkermansia*, *Lactobacillus* and *Bifidobacterium*, which have been shown to correlate negatively with clinical indicators of cognitive function [23]. Additionally, they may also have pathogenic relevance [46, 47]. Species-level microbiome profiling and metabolomic analyses would provide more granular insights into the role of the gut-brain axis in Alzheimer’s and other dementias.

Regional differences in *Bacteroides* abundance in AD indicate that diet and lifestyle have a crucial role in gut microbiome composition, which subsequently affects the gut-brain axis and development of AD. Our study highlights the relevance of region-based longitudinal studies in aging cohorts. The differences in *Bacteroides*, and the heterogeneity of other taxa, suggest the possibility of stratifying patient cohorts as a first step to microbiome-related interventions [31]. Moreover, the extent of changes in *Bacteroides*, one of the major constituents of the gut microbiome, is higher in the AD stage than the MCI stage. Along with the increase in *Phascolarctobacterium*, this underscores the need for early diagnosis at the MCI stage. Recent studies lend support to the possibility that non-drug interventions can be particularly impactful in this stage [4, 15, 16].

The strengths of this study include the comprehensive set of outcomes and rigorous methodology. Nonetheless, it is recognized that the interpretation of our study is subject to possible limitations. While most studies have documented medications taken by participants, only one study has addressed polypharmacy [17], which refers to the use of five or more daily medications, and is known to be detrimental to gut microbiome composition [48]. This may represent a source of bias in addition to observed heterogeneity in the gut microbiome composition of elderly individuals [49]. It would be informative to examine the differences in microbiome composition between participants who practice polypharmacy and those who don’t.

## Conclusion

In conclusion, we have discovered that the progression of Alzheimer’s is associated with more significant impact on species richness than evenness in the gut microbiome. We have obtained evidence that regional differences in diet and lifestyle can influence gut dysbiosis in AD patients. Our study has provided a glimpse into specific re-arrangements occur during the progression to AD. Therefore, we believe that our study will be helpful in the development of non-drug early-stage interventions which harness the power of the gut microbiome to affect the gut-brain axis.

## Supporting information

S1 Fig Publication bias detection. Funnel plots do not show any significant publication bias for Shannon index in AD, Shannon index in MCI and relative abundance of *Bacteroides*.
(PDF)

S1 Table PRISMA-P checklist. Preferred Reporting Items for Systematic review and Meta-Analysis Protocols 2015 checklist: recommended items to address in a systematic review protocol.
(PDF)

S2 Table Controlled vocabulary for search. List of controlled vocabulary terms used to formulate search queries.
(PDF)

S3 Table Study selection. List of full-text reports screened with reasons for exclusion.
(PDF)

S4 Table Overview of characteristics of included studies. Description of included studies, including cohort sizes, average age, proportion of female participants, diagnostic criteria, exclusion criteria, and ethics committee/review board approvals.
(PDF)

S5 Table Bioinformatic methods. A summary of reported taxonomic units and bioinformatic methods used for 16S/metagenomic data analysis in the included studies.
(PDF)

S6 Table PRISMA 2020 for abstracts checklist. (PDF)

S7 Table PRISMA 2020 checklist. (PDF)

S1 Appendix Diversity measures. Definition and units of *α*-diversity and *β*-diversity indices.
(PDF)

S2 Appendix Extended results. Additional results from meta-analysis of Simpson’s index, *β*-diversity measures and relative abundances of *Bifidobacteriaceae* and *Blautia*.
(PDF)

S1 Checklist PRISMA 2020 checklist. (PDF)

S2 Checklist PRISMA 2020 for abstracts checklist. (PDF)
