# Supplementary material for: Gut microbiome dysbiosis in Alzheimer’s disease and mild cognitive impairment: A systematic review and meta-analysis
Source: PLoS One. 2023 May 24;18(5):e0285346. doi: 10.1371/journal.pone.0285346 (PMC10208513; doi:10.1371/journal.pone.0285346)

S1 Figure. Publication bias detection

Funnel plots do not show any significant publication bias for A) Shannon index in AD, B) Shannon index in MCI and C) relative abundance of *Bacteroides*.

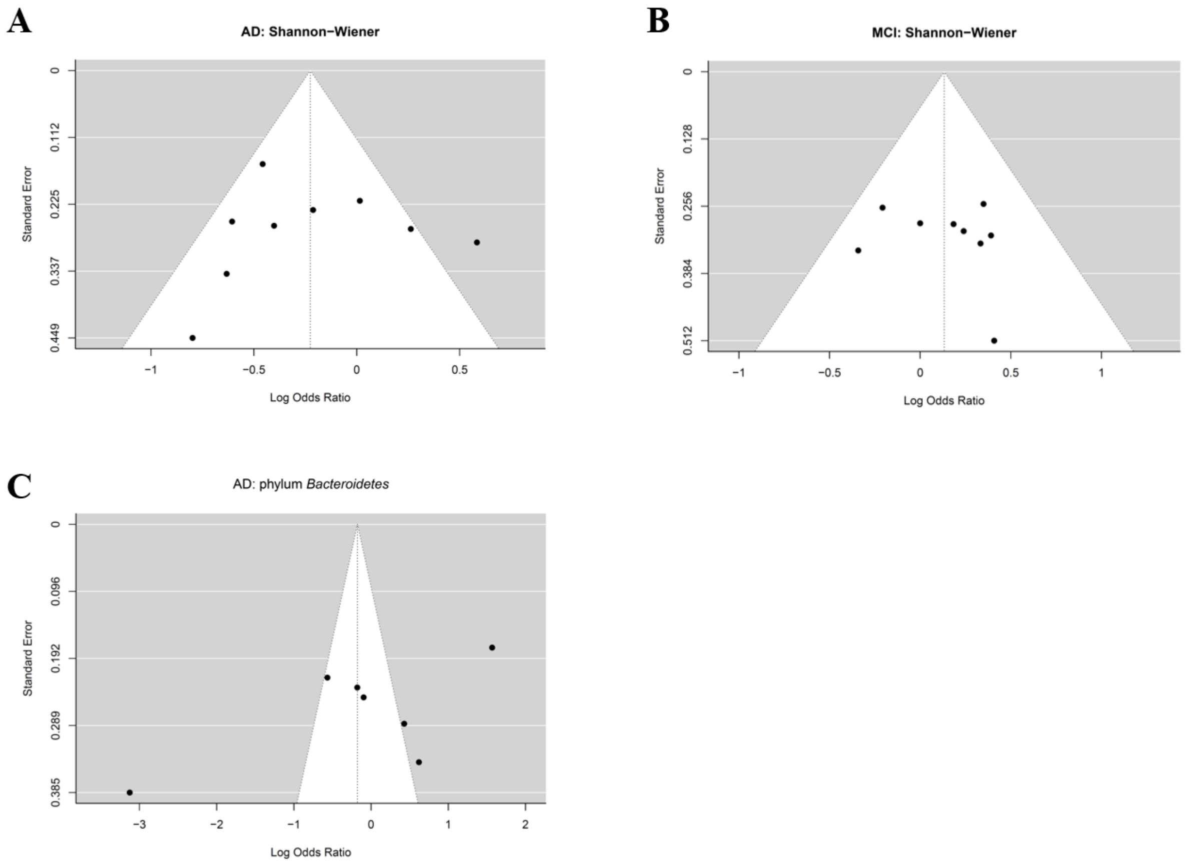

Supplement: S1 Fig — Funnel plots do not show any significant publication bias for Shannon index in AD, Shannon index in MCI and relative abundance of Bacteroides. (PDF) [file pone.0285346.s001.pdf]
