# Supplementary material for: Gut microbiome dysbiosis in Alzheimer’s disease and mild cognitive impairment: A systematic review and meta-analysis
Source: PLoS One. 2023 May 24;18(5):e0285346. doi: 10.1371/journal.pone.0285346 (PMC10208513; doi:10.1371/journal.pone.0285346)
Supplement: S1 Table — Preferred Reporting Items for Systematic review and Meta-Analysis Protocols 2015 checklist: recommended items to address in a systematic review protocol. (PDF) [file pone.0285346.s002.pdf]

S1 Table. PRISMA-P checklist

PRISMA-P (Preferred Reporting Items for Systematic review and Meta-Analysis Protocols) 2015 checklist: recommended items to address in a systematic review protocol

Section and topic	Item No	Checklist item
**ADMINISTRATIVE INFORMATION**		
Title:		
Identification	1a	Gut microbiome dysbiosis in Alzheimer's disease and mild cognitive impairment: A systematic review and meta-analysis
Update	1b	Not applicable
Registration	2	In accordance with the guidelines, our meta-analysis and systematic review protocol was registered with the International Prospective Register of Systematic Reviews (PROSPERO) on 10 May 2022, with registration number CRD42022328141.
Authors:		
Contact	3a	*Corresponding author: Aamna Mohammed AlShehhi aamna.alshehhi@ku.ac.ae<br>Author affiliation<br>Department of Biomedical Engineering, Khalifa University, Abu Dhabi, PO Box 127788, United Arab Emirates<br>- Email: Sherlyn Jemimah sherlyn.jemimah@ku.ac.ae - Chahd Maher Musthafa Chabib chahd.chabib@ku.ac.ae - Leontios Hadjileontiadis leontios.hadjileontiadis@ku.ac.ae
Contributions	3b	AMAS is the guarantor. AMAS and LH acquired funding, conceptualised the study and supervised the project. SJ developed the methodology for the meta-analysis (search strategy, selection criteria, data extraction methodology, statistical analysis) and performed the search, data extraction, analyses and visualization, and drafted the initial manuscript. AMAS was responsible for project administration and resources. AMAS and CMMC provided expert opinion and verified the data and methods. All authors read, provided feedback, participated in reviewing and editing, and approved the final manuscript.
Amendments	4	In the event of any amendments to the protocol, we will document the changes in the PROSPERO record CRD42022328141.
Support:		
Sources	5a	This meta-analysis and systematic review is funded by Khalifa University.
Sponsor	5b	Khalifa University is the sponsor, and no external funding has been received for this study. The authors declare no competing interests.
Role of sponsor or funder	5c	Khalifa University is funding the meta-analysis and systematic review. The funding supports and facilitates data management and analyses by the investigators. Khalifa University has no input on the interpretation or publication of the study results.
**INTRODUCTION**		
Rationale	6	Perturbations in gut microbiome composition, termed dysbiosis, has been linked to several diseases. In neurodegenerative disorders, the pathway between gut dysbiosis and neurodegeneration is marked by immune activation and inflammation. Case-control studies of Alzheimer's disease identify significant changes in gut microbial composition between healthy subjects and the disease cohort. However, till date and to the best of our knowledge, no specific microbial taxa have been consistently and uniquely associated with

		Alzheimer's. Further, case-control studies with smaller samples may be affected by low power and confounding factors. With several case-control studies made available in recent years, a meta-analysis and systematic review will help elucidate the direction and extent of gut dysbiosis in Alzheimer's disease.
Objectives	7	The meta-analysis and systematic review aims to determine the direction and extent of gut dysbiosis in Alzheimer's disease. To this end, the proposed protocol will answer the following question:<br>What are the gut microbiome markers of disease progression in human patients with mild cognitive impairment (MCI) or Alzheimer's disease (AD), compared to cognitively normal controls, in terms of gut microbiome diversity and relative taxon abundance?
**METHODS**		
Eligibility criteria	8	Eligible studies include case-control metagenomic and 16S studies of Alzheimer's disease (AD) and mild cognitive impairment (MCI) in humans with reported outcomes such as α-diversity, β-diversity ordination, relative abundances of various taxa and linear discriminant analysis effect sizes (LEfSe). Randomised controlled trial with reported outcomes prior to intervention are also considered, as are cohorts derived from longitudinal studies.<br>Patient inclusion criteria: Elderly human patients diagnosed with MCI or AD according to well-defined diagnostic criteria such as NIA/AA guidelines or DSM.<br>Exclusion criteria:<br>1. Antibiotic use within two weeks of sample collection<br>2. Presence of confounding conditions such as IBS, depression, cancer or any other genetic/neurological/gastrointestinal disorders<br>Exposure: Differences in gut microbiota between patients with mild cognitive impairment or Alzheimer's disease and cognitively normal elderly.<br>Control: Comparison groups consist of cognitively normal controls who are reasonably matched in age, gender and years of education, and follow a similar diet and lifestyle due to geographic proximity and common ethnicity.
Information sources	9	We searched literature databases, namely MEDLINE, Cochrane, EBSCO, EMBASE and Scopus. We did not search grey literature. We also scanned the reference lists of included studies and relevant reviews identified through the search.
Search strategy	10	We searched for papers in English published between Jan 1, 2010 and Mar 31, 2022. Search terms were generated using a controlled vocabulary provided in Table S2, by combining a term related to Alzheimer's with a term related to metagenomics. An example search query is "Alzheimer AND microbiome".
Study records:		
Data management	11a	Literature search results were imported into Rayyan for de-duplication and screening. Titles and abstracts were used for initial screening. Full-texted reports were assessed for eligibility.
Selection process	11b	Full-text copies of papers were assessed for eligibility by two authors, with any disagreements resolved by the corresponding author.
Data collection process	11c	Reported data in tabular and graphical form was extracted from full-text reports. The corresponding authors of included studies were contacted to request any missing data.
Data items	12	In addition to outcomes, the following data items are extracted:<br>1. Study location

		2. Size and mean age of the cohorts<br>3. Proportion of female participants<br>4. Diagnostic criteria used for AD and MCI<br>5. Exclusion criteria for participants<br>6. Sequencing and bioinformatic methods used for data analysis<br>7. Ethics committee/review board approvals
Outcomes and prioritization	13	The primary outcomes of interest are changes in α-diversity and changes in relative abundance of various taxa. For our meta-analysis, our diversity outcomes include Shannon-Wiener index, Simpson's index, Chao index, abundance-based coverage estimators (ACE), and number of species observed (Sobs).<br>Additionally, we perform a qualitative synthesis of β-diversity indices (Bray-Curtis, Jaccard, weighted/unweighted UniFrac, Aitchison distances) and LEfSe.
Risk of bias in individual studies	14	The risk of bias for each study was assessed using criteria appropriate for the selected study designs.<br>Risk of bias was assessed for the following items:<br>1. Study design and objectives<br>2. Selection of participants and constitution of study groups<br>3. Other information bias<br>4. Statistical methods to control confounding<br>5. Statistical methods excluding methods to control confounding<br>6. Conflict of interest<br>7. Summary risk-of-bias assessment
Data synthesis	15a	Primary outcomes, ie. α-diversity and relative abundances were quantitatively analysed.
	15b	For each outcome, we will combine the studies with a random-effects model using the DerSimonian and Laird (1986) implementation using the metafor package in R. We present the results using forest plots for each outcome of interest, with the weight (denoted by square size) indicating the influence of an individual study on the pooled result. Inconsistency between groups is quantified using the I^2 statistic, which is the total variation that is attributed to the true difference between the studies, with values greater than 50% indicating substantial heterogeneity.
	15c	Subgroup analyses were performed for outcomes with significant heterogeneity and sufficient number of reporting studies.
	15d	Qualitative synthesis was performed for secondary outcomes, namely β-diversity and LEfSe.
Meta-bias(es)	16	Funnel plots were assessed for outcomes with seven or more reporting studies to detect publication bias.
Confidence in cumulative evidence	17	The strength of the evidence was not formally assessed as the outcome data is generated from next-generation sequencing methods and analysed using standard bioinformatics workflows.

Reference: Shamseer L, Moher D, Clarke M, Ghersi D, Liberati A, Petticrew M, Shekelle P, Stewart L, PRISMA-P Group. Preferred reporting items for systematic review and meta-analysis protocols (PRISMA-P) 2015: elaboration and explanation. BMJ 2015; 349(jan02 1): g7647. doi: 10.1136/bmj.g7647
