# Supplementary material for: Gut microbiome dysbiosis in Alzheimer’s disease and mild cognitive impairment: A systematic review and meta-analysis
Source: PLoS One. 2023 May 24;18(5):e0285346. doi: 10.1371/journal.pone.0285346 (PMC10208513; doi:10.1371/journal.pone.0285346)
Supplement: S2 Table — List of controlled vocabulary terms used to formulate search queries. (PDF) [file pone.0285346.s003.pdf]

S2 Table. Controlled vocabulary for search

List of controlled vocabulary terms used to formulate search queries.

Related to disease	Related to metagenomics and the microbiome
Alzheimer	metagenomic
dementia	microbiota
MCI	microbiome
mild cognitive impairment	gut brain axis
	16S

Search queries were generated by combining a term related to Alzheimer's with a term related to metagenomics.
