# Supplementary material for: Gut microbiome dysbiosis in Alzheimer’s disease and mild cognitive impairment: A systematic review and meta-analysis
Source: PLoS One. 2023 May 24;18(5):e0285346. doi: 10.1371/journal.pone.0285346 (PMC10208513; doi:10.1371/journal.pone.0285346)
Supplement: S3 Table — List of full-text reports screened with reasons for exclusion. (PDF) [file pone.0285346.s004.pdf]

S3 Table. Study selection

List of full-text reports screened with reasons for exclusion.

Authors	Year	DOI/PubMed	Inclusion/Exclusion (reason)
Duan et al.	2021	10.3389/fcimb.2021.592842	Included
Guo et al.	2021	10.3233/jad-201040	Included
Haran et al.	2019	10.1128/mBio.00632-19	Included
Hou et al.	2021	10.3389/fnins.2021.619051	Included
Khine et al.	2020	10.18632/aging.202277	Included
Li et al.	2019	10.1016/j.jalz.2019.07.002	Included
Ling et al.	2021	10.3389/fcell.2020.634069	Included
Liu et al.	2021	10.1111/cns.13451	Included
Liu et al.	2019	10.1016/j.bbi.2019.05.008	Included
Nagpal et al.	2020	10.1016/j.ebiom.2020.102950	Included
Pan et al.	2021	10.1155/2021/5578958	Included
Ueda et al.	2021	10.1016/j.xcrm.2021.100398	Included
Vogt et al.	2017	10.1038/s41598-017-13601-y	Included
Xi et al.	2021	10.1186/s12866-021-02286-z	Included
Yildirim et al.	2022	10.1128/msystems.00004-22	Included
Zhou et al.	2021	10.3233/jad-201497	Included
Zhuang et al.	2018	10.3233/jad-180176	Included
Jiang et al.	2021	PMID 34017407	Excluded (Uncontrolled pilot study)
Suhocki et al.	2022	10.1002/trc2.12225	Excluded (Uncontrolled pilot study)
Rowan-Nash et al.	2020	10.1016/j.isci.2020.100905	Excluded (Samples from AD patients not demarcated)
Sheng et al.	2021	10.3233/jad-210259	Excluded (Samples from AD patients not demarcated)
Stadlbauer et al.	2020	10.1186/s12877-020-01644-2	Excluded (Samples from AD patients not demarcated)
Haeger et al.	2020	10.1002/trc2.12032	Excluded (Results not published yet)
Jiang et al.	2021	10.1002/alz.12171	Excluded (Results not published yet)
Prinelli et al.	2020	10.1186/s12877-020-01652-2	Excluded (Results not published yet)
Yue et al.	2019	10.1186/s13063-019-3607-x	Excluded (Results not published yet)
Wu et al.	2021	10.3390/nu13010228	Excluded (Patient dataset overlap)
Saji et al.	2021	10.1016/j.jstrokecerebrovasdis.2020.1055 68	Excluded (Non-NGS sequencing)
Saji et al.	2019	10.1038/s41598-018-38218-7	Excluded (Non-NGS sequencing)
Saji et al.	2019	10.1038/s41598-019-55851-y	Excluded (Non-NGS sequencing)
Saji et al.	2020	10.1038/s41598-020-65196-6	Excluded (Non-NGS sequencing)
Saji et al.	2022	10.1016/j.nut.2021.111524	Excluded (Non-NGS sequencing)
Ivakhniuk T & Ivakhniuk Y	2021	PMID 34103438	Excluded (Metagenomics not performed on AD group)
Qian et al.	2020	10.1093/brain/awaa201	Excluded (Metagenomics not performed on AD group)
Ling et al.	2021	10.3389/fcell.2020.631460	Excluded (Fungal microbiome)
Askarova et al.	2021	0.1002/alz.052741	Excluded (Full-text report not available)
Geng M	2021	10.1002/alz.055865	Excluded (Full-text report not available)
Marizzoni et al.	2021	10.1002/alz.053455	Excluded (Full-text report not available)
Mombelli et al.	2021	10.1002/alz.042737	Excluded (Full-text report not available)
Saha et al.	2021	10.2174/1567205018666210907160854	Excluded (Full-text report not available)
Saji N	2020	10.11477/mf.1416201513	Excluded (Full-text report not available in English)
Zhang et al.	2021	10.1093/ajcn/nqab078	Excluded (Full-text report not available)
