# Supplementary material for: Gut microbiome dysbiosis in Alzheimer’s disease and mild cognitive impairment: A systematic review and meta-analysis
Source: PLoS One. 2023 May 24;18(5):e0285346. doi: 10.1371/journal.pone.0285346 (PMC10208513; doi:10.1371/journal.pone.0285346)
Supplement: S4 Table — Description of included studies, including cohort sizes, average age, proportion of female participants, diagnostic criteria, exclusion criteria, and ethics committee/review board approvals. (PDF) [file pone.0285346.s005.pdf]

S4 Table. Overview of characteristics of included studies

Study	Location	AD			MCI			Cognitively normal			Diagnostic criteria	Exclusion criteria	Ethics committee/ Review board approval
		N	Age (SD)	%F	N	Age (SD)	%F	N	Age (SD)	%F			
Duan et al., 2021	Beijing, China				18	71.22 (3.98)	87.5	36	70.78 (4.41)	72.2	DSM-IV criteria for dementia and ADNI2 criteria for classification of aMCI and CN	Not provided	Ethics Committee of XuanWu Hospital at Capital Medical University, Beijing, China
Guo et al., 2021	Guangzhou, China	18	63.5 (4.7)	88.9	20	64.5 (4.5)	80	18	64.2 (4.7)	77.8	2018 National Institute of Aging and Alzheimer's Association (NIA-AA) guidelines	Family history of dementia; any other neurodegenerative diseases (eg. Parkinson's, ALS) or mental illness (eg schizophrenia); severe cardiac, pulmonary, hepatic, intestinal or renal diseases; any tumour; history of antibiotic or glucocorticoid use within six months	Ethics Committee of Sun Yat-sen Memorial Hospital, Sun Yat-sen University, Guangzhou, China
Haran et al., 2019	Massachusetts, USA	24	84.7 (8.1)	83.3				51	83.0 (10.2)	84.3	Diagnosis of Alzheimer's disease was made by querying the facility medical record and confirmed by the facility treating physician	Diarrheal illness or antimicrobial exposure in the last 4 weeks; dysphagia; feeding tube use	Institutional review board (IRB) at the University of Massachusetts Medical School (docket H00010892)
Hou et al., 2021	Chengdu, China	30	71.9 (6.9)	43.3				47	71.1 (6.7)	53.2	DSM-III, for revised dementia criteria; National Institute of Neurological and Communicative Disorders and Stroke and the Alzheimer Disease and Related Disorders Association criteria	Any significant neurologic or gastrointestinal disease, chronic constipation, *C. difficile* infection, history of alcohol/substance dependence, psychiatric disorders, any cancers; antibiotics use 3 months prior to sampling; eating disorder; dietary change in the past month; nutritional supplement (probiotics or special diet); history of	Ethics Committee of School of Public Health at Shanghai Jiao Tong University for Human Subject Research, Shanghai, China

Study	Location	AD			MCI			Cognitively normal			Diagnostic criteria	Exclusion criteria	Ethics committee/ Review board approval
		N	Age (SD)	%F	N	Age (SD)	%F	N	Age (SD)	%F			
												gastrointestinal operations which can confound results	
Khine et al., 2020	Singapore				46	67 (range 60-79)	69.6	77	65 (range 60-85)	75.3	MCI diagnostic criteria: 1) at least, one age-education adjusted neuropsychological test Z score less than (-)1.5; 2) did not meet Diagnostic and Statistical Manual of Mental Disorders (DSM-V) criteria for dementia; 3) had memory/cognitive complaints, preferably corroborated by a reliable informant; 4) had intact activities of daily living and functioned independently	Neurological conditions (eg., epilepsy, Parkinson's); psychiatric conditions (major depressive disorder); significant visual or hearing impairment, colour blindness, upper and lower limb motor difficulties, terminal illness	Institutional Review Board of the National University of Singapore, Singapore (NUS-IRB Ref: Nos. 10-517, 13-168 and B14-110); study registered at ClinicalTrials.gov (NCT02286791)
Li et al., 2019	Shanghai, China	30	66.3 (5.1)	50	30	65.4 (7.6)	60	30	63.9 (5.1)	56.7	National Institute on Aging-Alzheimer's Association (NIA-AA) workgroup criteria with global score of Clinical Dementia Rating (CDR) of 0.5 for MCI, and ≥1 for AD diagnosis	Evidence of stroke, major mood problems, other neurodegenerative causes of dementia, HIV or serious bacterial infection in the medical history; received antibiotics or blood transfusion recently	Ethics committee approval information not provided. The study was conducted at the Ruijin Hospital affiliated to Shanghai Jiao Tong University School of Medicine, China
Ling et al., 2021	Hangzhou, China	100	74.14 (9.21)	57				71	73.1 (7.75)	50.7	National Institute of Neurological and Communicative Diseases and Stroke/AD and Related Disorders Association (year not specified)	Family history of dementia; other neurodegenerative diseases; mental illness; tumours; antibiotic, prebiotic, probiotic, or synbiotic use in the past month; active viral/bacterial/fungal infections; any other disease (eg. IBD, IBS, autoimmune conditions)	Ethics Committee of Lishui Second People's Hospital, Zhejiang, China

Study	Location	AD			MCI			Cognitively normal			Diagnostic criteria	Exclusion criteria	Ethics committee/ Review board approval
		N	Age (SD)	%F	N	Age (SD)	%F	N	Age (SD)	%F			
Liu et al., 2019	Hangzhou, China	33	74.85 (11.37)	42.4	32	70.53 (11.0)	56.3	32	76.88 (9.35)	50	DSM-IV (1994) and 2007 guidelines of the National Institute of Neurological and Communicative Disorders and the Stroke and Alzheimer Disease and Related Disorders Association (NINCDS-ADRDA)	Other causes of cognitive impairment; history of antibiotics, probiotics, prebiotics, or synbiotics use within two months; severe malnutrition, infection, drug or alcohol addicts; IBS/IBD in the last year; schizophrenia, schizoaffective disorder or primary affective disorder; heart, brain, liver, kidney and hematopoietic diseases; severe auditory, visual or motor deficits that may interfere with cognitive testing	Ethics committee of the First affiliated hospital, School of Medicine, Zhejiang University, Hangzhou, China
Liu et al., 2021	Hangzhou, China				20	68.8 (11.2)	60	22	72.7 (8.05)	56.7	Petersen criteria for MCI*	Other causes of cognitive impairment; history of antibiotics, probiotics, prebiotics, or synbiotics use within two months; severe malnutrition, infection, drug or alcohol addicts; IBS/IBD in the last year; schizophrenia, schizoaffective disorder or primary affective disorder; heart, brain, liver, kidney and hematopoietic diseases; severe auditory, visual or motor deficits that may interfere with cognitive testing; MR incompatibility	Ethics Committee of the First Affiliated Hospital, College of Medicine, Zhejiang University

Study	Location	AD			MCI			Cognitively normal			Diagnostic criteria	Exclusion criteria	Ethics committee/ Review board approval
		N	Age (SD)	%F	N	Age (SD)	%F	N	Age (SD)	%F			
Nagpal et al., 2019	North Carolina, USA				11	64.3 (7.7)	72.7	6	65.2 (3.7)	66.7	ADNI2 criteria for early MCI	Other neurodegenerative illness; history of stroke, epilepsy, seizure; focal brain lesions or head injury with loss of consciousness; satisfies DSM-IV criteria for other major psychiatric disorders; alcohol or substance abuse; visual/auditory impairment; diabetes; use of cholesterol/lipid lowering medications; clinically significant elevations in liver function tests; cancer; contraindications for MRI; significant medical illness or organ failure; use of anticonvulsants (other than cholinesterase inhibitors or memantine); regular use of narcotic analgesics; major digestive disorders, absorption issues, or surgeries that may be exacerbated by diet changes	Institutional Review Boards of the Wake Forest School of Medicine, Winston-Salem, NC, United States; trial registered prior to the recruitment (Clinical Trials # NCT02984540)
Pan et al., 2021	Changsha , China				22	71.45 (8.03)	63.6	26	67.31 (5:27)	73.1	Diagnostic criteria not provided; report differences in terms of MMSE and ADL scores	Antibiotics use in the last 6 months; probiotics in the last 3 months; gastrointestinal disorders (eg., chronic diarrhoea, IBD, infectious gastroenteritis); gastrointestinal surgery in the past three years	Ethics Committee of The Third Xiangya Hospital of Central South University, Changsha, China
Ueda et al., 2021	Kusatsu, Japan	7	83.0 (9.3)	71.4	15	79.8 (6.1)	60	21	72.7 (5.3)	61.9	Diagnostic and Statistical Manual of Mental Disorders (4th edition) criteria for AD;	Antibiotic use w2 weeks prior to sampling; GI tract surgery in the past 6 months; history of GI disorders such as IBD,	Ethics committee of the Tokyo Metropolitan Institute of Gerontology (TMIG)

Study	Location	AD			MCI			Cognitively normal			Diagnostic criteria	Exclusion criteria	Ethics committee/ Review board approval
		N	Age (SD)	%F	N	Age (SD)	%F	N	Age (SD)	%F			
											Petersen criteria for MCI*	IBS, GI cancer or gastrostomy; diabetes; significant neurologic or psychiatric diseases other than AD	
Vogt et al., 2017	Wisconsin, USA	25	71.3 (7.3)	68				25	69.3 (7.5)	72	NINCDS/ADRDA criteria	Any significant neurologic disease, history of alcohol/substance dependence, major psychiatric disorders (depression), or any other significant medical illness; antibiotics use in the past 6 months; corticosteroid use; immune stimulating medications; immunosuppressive agents; probiotics; major dietary change during previous month; major GI tract surgery in past 5 years with the exception of cholecystectomy and appendectomy; major bowel resection; active uncontrolled GI disorders or diseases including IBD, indeterminate colitis, IBS, infectious gastroenteritis, colitis or gastritis, persistent or chronic diarrhoea, *C. difficile* or *H. pylori* infection, or chronic constipation	The University of Wisconsin Health Science Institutional Review Board
Xi et al., 2021	Shanghai, China	21	76.2 (IQR 9.9)	38.1				44	78.4 (IQR 6.6)	54.6	Consensus diagnosis of AD based on DSM-IV and NINCDS-ADRDA criteria	Use of antibiotics, probiotics, or prebiotics within a month before sampling; corticosteroid use, immune stimulating medications and immunosuppressive agents; use of anti-	Ethics Committee of the Department of Public Health at Fudan University (IRB#2019-04-0739) and the Medical Ethics Committee of Huashan Hospital at

Study	Location	AD			MCI			Cognitively normal			Diagnostic criteria	Exclusion criteria	Ethics committee/ Review board approval
		**N**	**Age (SD)**	**%F**	**N**	**Age (SD)**	**%F**	**N**	**Age (SD)**	**%F**			
												depressant; gastrointestinal surgery in past 5 years; HIV or serious bacterial infection in the medical history; IBD, persistent, infectious gastroenteritis, colitis or gastritis; diarrhoea or constipation at sampling	Fudan University (IRB#2009-195), Shanghai, China
Yildirim et al., 2022	Kayseri and Istanbul, Turkey	47	71.4 (5.1)	48.9	27	69.2 (6.4)	40.7	51	67 (5.3)	45	National Institute on Aging-Alzheimer's Association Workgroups on diagnostic guidelines for Alzheimer's disease	History of substance abuse, any significant neurologic disease, and psychiatric disorders such as major depression; probiotics or antibiotics use during the study period or within 1 month prior to providing a sample or who had undergone major gastrointestinal (GI) tract surgery in past 5 years.	The Istanbul Medipol University and Erciyes University Ethical Review Boards approved this study (approval no. 186/16.4.2015 and 85/20.02.2015)
Zhou et al., 2021	Beijing, China	60	72.82 (7.25)	60				32	71.06 (5.92)	56.3	Core clinical criteria of probable AD in the revised National Institute on Aging-Alzheimer's Association (NIA-AA) diagnostic guidelines for AD	Family history of dementia; history of stroke or other distinct neurological diseases (such as brain tumours, Parkinson's, MS, epilepsy, brain trauma, normal intracranial pressure hydrocephalus); severe medical diseases (such as cardiac insufficiency, COPD, liver and kidney insufficiency, abnormal thyroid function, and infectious disease); mental illness such as major depression; systemic antibiotics or probiotics use within 3 months of faecal sample collection; use of	Ethics Committee of Xuanwu Hospital of Capital Medical University

Study	Location	AD			MCI			Cognitively normal			Diagnostic criteria	Exclusion criteria	Ethics committee/ Review board approval
		N	Age (SD)	%F	N	Age (SD)	%F	N	Age (SD)	%F			
												corticosteroids, immunostimulating drugs, and/or immunosuppressive agent; 7) history of gastrointestinal and bowel resection surgery except for cholecystectomy; gastrointestinal diseases, including IBS, colitis or gastritis, chronic diarrhoea, or constipation	
Zhuang et al., 2018	Chongqing, China	43	70.12 (8.78)	46.5				43	69.72 (9.24)	46.5	National Institute of Neurological and Communicative Diseases and Stroke/AD and Related Disorders Association (year not specified)	Family history of dementia; any kind of other neurodegenerative disease (eg, Parkinson's, ALS); severe cardiac, pulmonary, hepatic, renal diseases, or any kind of tumour; enduring mental illness (eg, schizophrenia); history of taking antibiotics within six months, intestinal diseases (eg, IBS)	Institutional Review Board of Daping Hospital, Military Medical University, Chongqing, China

Abbreviations %F: Percentage of female participants, ALS: Amyotrophic Lateral Sclerosis, CNS: Central Nervous System, COPD: Chronic Obstructive Pulmonary Disease, DSM: Diagnostic and Statistical Manual of Mental Disorders, GI: Gastrointestinal, HIV: Human Immunodeficiency Virus, HRT: Hormone Replacement Therapy, IBD: Infectious Bowel Disease, IBS: Irritable Bowel Syndrome, MR: Medication Reconciliation, MRI: Magnetic Resonance Imaging, MS: Multiple sclerosis

*****Petersen RC. Mild cognitive impairment as a diagnostic entity. J Intern Med. 2004 Sep;256(3):183–94. doi: 10.1111/j.1365-2796.2004.01388.x
