# Supplementary material for: Gut microbiome dysbiosis in Alzheimer’s disease and mild cognitive impairment: A systematic review and meta-analysis
Source: PLoS One. 2023 May 24;18(5):e0285346. doi: 10.1371/journal.pone.0285346 (PMC10208513; doi:10.1371/journal.pone.0285346)
Supplement: S5 Table — A summary of reported taxonomic units and bioinformatic methods used for 16S/metagenomic data analysis in the included studies. (PDF) [file pone.0285346.s006.pdf]

S5 Table. Bioinformatic methods

A summary of reported taxonomic units and bioinformatic methods used for 16S/metagenomic data analysis in the included studies.

Study	Experimental data	Details of bioinformatics methods
Duan et al., 2021	V3-V4 regions of the 16S rRNA	All reads were grouped into OTUs at sequence identity 97% using QIIMEv2.0 against Greengenes database v135. OTUs filtering threshold was set at 0.1%. Taxonomic assignment was performed with RDP classifier against the Silva (SSU128) 16S rRNA database with a confidence threshold of 70%. Bacterial diversity was determined by α-diversity (Chao1, Shannon's index, InvSimpson index) and β-diversity (PCA, PCoA). LEfSe was performed among NC, SCD and aMCI groups.
Guo et al., 2021	V3-V4 regions of the 16S rRNA	Tags were clustered at 97% sequence identity using UCLUST of QIIME v2.0 against Greengenes database v13.8 to obtain OTUs. OTUs were taxonomically classified using the same database. The filtering threshold was set at 0.005%. α-diversity (evenness, faith pd, Shannon index) and β-diversity (Bray Curtis, unweighted and weighted UniFrac, and PCA) were investigated with QIIME. LEfSe was performed.
Haran et al., 2019	Shotgun metagenomics reads	Reads were profiled for microbial species relative abundances by mapping them to a NCBI bacterial genomes k-mer database with Kraken and by reconstructing the resulting relative abundance profile at the species level with Bracken. Normalized taxonomic abundances were then used for downstream statistical analysis in R. β-diversity was evaluated using Jaccard distances and visualized using t-SNE.
Hou et al., 2021	V3-V4 regions of the 16S rRNA	Sequences were clustered into OTUs using UPARSE v7.1 with 97% of similarity and taxonomically classified using the Greengenes database v13.5. Bacterial diversity was determined by α-diversity (Shannon's index) and β-diversity (unweighted/weighted UniFrac, Bray-Curtis distances and PCoA). LEfSe was also performed.
Khine et al., 2020	V3-V4 regions of the 16S rRNA	Sequences were clustered into OTUs with 97% of similarity and taxonomically classified using the Greengenes database v13.8. Among bacterial genera, only the top 1% of relative abundances were used. Bacterial diversity was investigated using α-diversity (Chao1 and Shannon) and β-diversity (Bray-Curtis distances and PCoA).
Li et al., 2019	V3-V4 regions of the 16S rRNA	Rarefied OTUs were used to calculate α-diversity (Chao1, faith pd, S_{obs}, and Shannon), β-diversity (weighted and unweighted Unifrac) and LEfSe scores.
Ling et al., 2021	V3-V4 regions of the 16S rRNA	Sequences were processed using QIIMEv1.9. OTUs were picked with USEARCH v7 against Greengenes v13.8 at 97% similarity. The filtering threshold was set at 0.005%. Taxonomic assignment was performed using RDP Classifier against Greengenes v13.8. Diversity was measured using α-diversity indices (ACE, Chao1, Shannon, Simpson, evenness, and PD whole tree) and β-diversity (Jaccard, Bray-Curtis, unweighted and weighted Unifrac, PCoA). Differences in abundance were measured using LEfSe.
Liu et al., 2019	V3-V4 regions of the 16S rRNA	Sequences were clustered into OTUs at 97% similarity and taxonomic assignment was done with QIIME v1.8 against Greengenes v13.8. Bacterial diversity was determined by α-diversity (Shannon, Simpson, Chao1, ACE) and β-diversity (PCoA). LEfSe method was used to characterize the taxa with statistical significance.
Liu et al., 2021	V3-V4 regions of the 16S rRNA	All reads were grouped into OTUs at a sequence identity of 97%. The 16S rRNA reads were processed and compared using QIIME v1.8.0 to calculate α-diversity (Chao1, ACE, Shannon and Simpson).
Nagpal et al., 2019	V4 regions of the 16S rRNA	Sequences were clustered into OTUs at 97% similarity and taxonomic assignment was done with QIIME v1.9.1 against Greengenes database. OTUs were rarefied. Bacterial diversity was determined by α-diversity (PD whole tree, Shannon, Sobs, Chao1) and β-diversity (unweighted and weighted UniFrac, PCoA). LEfSe method was used to characterize the taxa with statistical significance between CN and MCI as well as dietary conditions.
Pan et al., 2021	V1-V9 regions of the 16S rRNA	Sequences were processed with QIIME. Bacterial diversity was determined by α-diversity (ACE, Shannon, Simpson, Chao1) and β-diversity (NMDS, PCoA). LEfSe method was used to characterize the taxa with statistical significance. Taxonomic units were not mentioned.
Ueda et al., 2021	V3-V4 regions of the 16S rRNA and shotgun reads	Sequences were processed using QIIMEv2 and taxonomic assignment was performed with a naïve Bayes classifier against the Genome Taxonomy Database (GTDB, July 2019). The filtering threshold was set at 0.01%. Diversity was measured using α-diversity indices (ACE, Chao1, Shannon, Simpson, evenness, and PD whole tree) and β-diversity (Jaccard, Bray-Curtis, unweighted and weighted Unifrac, PCoA).
Vogt et al., 2017	V3-V4 regions of the 16S rRNA	Sequences were processed using mothur v1.39.1. Sequences were aligned to the SILVA 16S rRNA database, clustered with 97% similarity into OTUs and taxonomically assigned using a naïve Bayes classifier against Greengenes v13.8. Richness (ACE, Chao1), α-diversity (InvSimpson, Shannon, Faith PD) and β-diversity (Bray-Curtis, unweighted and weighted UniFrac) metrics were calculated.
Xi et al., 2021	V3-V4 regions of the 16S rRNA	Sequences were processed and clustered into OTUs at 97% similarity using UPARSE v7.1. Taxonomies were assigned using RDP Classifier with at least 70% confidence against the Silva (SSU132) 16S rRNA database. Diversity was measured using α-diversity indices (ACE, Shannon, InvSimpson) and β-diversity (Bray-Curtis and PCoA). Statistically significant differences in

Study	Experimental data	Details of bioinformatics methods
		specific bacterial taxa were analyzed using LEfSe.
Yıldırım et al., 2022	V3-V4 regions of the 16S rRNA	Sequences were processed in the DADA2 pipeline to obtain rarefied and unrarefied ASVs. Diversity was measured using α-diversity indices (Chao1, Shannon, InvSimpson) and β-diversity (Bray-Curtis, Jaccard and PCA).
Zhou et al., 2021	V3-V4 regions of the 16S rRNA	Sequences were processed and clustered into OTUs at 97% similarity using UPARSE. OTUs were classified using mothur against the Ribosomal Database Project (RDP). Diversity was measured using α-diversity indices (Chao1, ACE, S_{obs}, Shannon, Simpson) and β-diversity (Bray-Curtis, Jaccard, weighted/unweighted UniFrac and PCoA). LEfSe analysis was performed to identify the differences in bacterial taxa between the groups.
Zhuang et al., 2018	V3-V4 regions of the 16S rRNA	Sequences were clustered with 97% similarity into OTUs and classified using RDP. Diversity was measured using α-diversity indices (Chao1, S_{obs}, Shannon, Simpson) and β-diversity (UniFrac, PCoA). LEfSe analysis was performed.
