# Supplementary material for: Gut microbiome dysbiosis in Alzheimer’s disease and mild cognitive impairment: A systematic review and meta-analysis
Source: PLoS One. 2023 May 24;18(5):e0285346. doi: 10.1371/journal.pone.0285346 (PMC10208513; doi:10.1371/journal.pone.0285346)
Supplement: S1 Appendix — Definition and units of α-diversity and β-diversity indices. (PDF) [file pone.0285346.s009.pdf]

S1 Appendix. Diversity measures

The definition and units of various α-diversity and β-diversity indices[1,2] are provided below.

Shannon-Wiener index

Alpha-diversity is a measure of local diversity. Th Shannon-Wiener index, based on the Shannon entropy formula, and is a measure of both the richness (number of species) and evenness (distribution of each species):

$$H = -\sum p_i \times \ln p_i$$

where p_i is the relative abundance of a species *i*.

Simpson's index

Simpson's index is based on the probability of two individuals sampled from a population belonging to the same species. The formula is

$$D = \frac{\sum_1^k n_i(n_i - 1)}{n(n-1)}$$

where n_i refers to the number of individuals from a sample belonging to species *i*, in a total population of size *n* containing *k* species. A highly even population will have lower values of Simpson's index.

Species observed (S_{obs})

This index refers to the total number of species observed in a sample and provides no indication of species distribution or evenness.

Chao index

The Chao index uses the number of species observed and adds a correction factor incorporating the number of species represented by one or two individuals (ie, rare species). The formula is

$$S_{Chao} = S_{obs} + \frac{f_1^2}{2f_2}$$

where S_{obs} is the number of observed species, f_1 is the number of species represented by one individual and f_2 is the number of species represented by exactly two individuals.

Abundance-based Coverage Estimators (ACE)

For a given sample, with S_{rare} being the number of species with less than 10 individuals, S_{abund} being the number of species with more than 10 individuals and n_{rare} being total number individuals in rare species, the formula for ACE is

$$S_{ACE} = S_{abund} + \frac{S_{rare}}{C_{ACE}} + \frac{f_1}{C_{ACE}}\gamma_{ACE}^2$$

where C_{ACE} is the sample coverage estimate given by

$$C_{ACE} = 1 - \frac{f_1}{n_{rare}}$$

and γ_{ACE}^2 is the coefficient of variation given by

$$\gamma_{ACE}^2 = max\left[\frac{S_{rare}}{C_{ACE}}\frac{\sum_{k=1}^{10} k(k-1)f_k}{(n_{rare})(n_{rare}-1)} - 1, 0\right]$$

Bray-Curtis distance

Beta-diversity indices are used to quantify the difference between the reference (ie, healthy controls) and samples (from patients) in terms of abundance. The formula for Bray-Curtis distance is

$$d_{ij} = \frac{\sum_{k=1}^{n}\left|x_{i,k} - x_{j,k}\right|}{\sum_{k=1}^{n}(x_{i,k} + x_{j,k})}$$

where d_{ij} is the Bray-Curtis distance between two samples *i* and *j*, and *x* is a vector of the relative abundances of *k* species. The term $x_{i,k}$ refers to the abundance of species *k* in sample *i*. The distance ranges from 0 to 1, where 0 indicates complete similarity and 1 indicates complete dissimilarity.

Jaccard distance

The Jaccard distance is based on the number of species present in both the reference and sample. The formula is

$$d_{ij} = 1 - \frac{\left|S_i \cap S_j\right|}{\left|S_i \cup S_j\right|}$$

where the numerator is the number of species present in both samples, and the denominator is the total number of species present in the samples. S_i and S_j represent the species present in samples *i* and *j*, respectively.

UniFrac distance[3]

UniFrac distance considers phylogenetic information to compare the gut microbial compositions of two samples. In unweighted UniFrac, the distance is calculated using the fraction of branch lengths unique to either community in the phylogenetic tree. In weighted UniFrac, the branch lengths are weighted with the difference in abundance. The formula for UniFrac distance (given a phylogenetic tree) is

$$W = \frac{\sum_{i=1}^{N} I_i \left|\frac{A_i}{A_T} - \frac{B_i}{B_T}\right|}{\sum_{j=1}^{S} L_j}$$

where *N* is the number of nodes in the tree, *S* the number of sequences represented, A_T and B_T the total number sequences in samples A and B, A_i and B_i the number of sequences from samples A and B in node *i*, I_i the branch length between the node and its parent, and L_j the total branch length between sequence *j* from root to tip.

Aitchison distance[4,5]

The Aitchison distance metric is based on a centered log-ratio transformation of the composition data from the samples. The formula for two samples *x* and *y* is

$$\Delta(x, y) = \sqrt{\sum_{i=1}^{D}\left\{\ln\frac{x_i}{g(x)} - \ln\frac{y_i}{g(y)}\right\}^2}$$

where *g(x)* denotes the geometric mean $(x_1 \ldots x_D)^{1/D}$.

References

1. Magurran AE. Measuring biological diversity. 2nd ed. Oxford, U.K: Blackwell Science Ltd; 2004.
2. Jost L. Entropy and diversity. Oikos 2006; 113: 363–375.
3. Lozupone C, Knight R. UniFrac: a new phylogenetic method for comparing microbial communities. Appl Environ Microbiol 2005; 71: 8228–8235. doi: 10.1128/AEM.71.12.8228-8235.2005
4. Aitchison J et al. Logratio analysis and compositional distance. Math Geol 2000; 32: 271–275.
5. Quinn TP, Erb I, Richardson MF, Crowley TM. Understanding sequencing data as compositions: an outlook and review. Bioinformatics 2018; 34: 2870–2878. doi: 10.1093/bioinformatics/bty175
