# Supplementary material for: Gut microbiome dysbiosis in Alzheimer’s disease and mild cognitive impairment: A systematic review and meta-analysis
Source: PLoS One. 2023 May 24;18(5):e0285346. doi: 10.1371/journal.pone.0285346 (PMC10208513; doi:10.1371/journal.pone.0285346)
Supplement: S2 Appendix — Additional results from meta-analysis of Simpson’s index, β-diversity measures and relative abundances of Bifidobacteriaceae and Blautia. (PDF) [file pone.0285346.s010.pdf]

S2 Appendix. Extended results

Four studies have reported Simpson's index for AD[1-3] and MCI[1,2,4]. No significant changes were observed in AD (SMD -0·04, 95% CI -0·4 to 0·33; p=0·83; figure S2). In MCI, there was an increase approaching significance (SMD 0·57, 95% CI -0·06 to 1·21; p=0·07), with significant, high heterogeneity (I^2 = 73%; p=0·03).

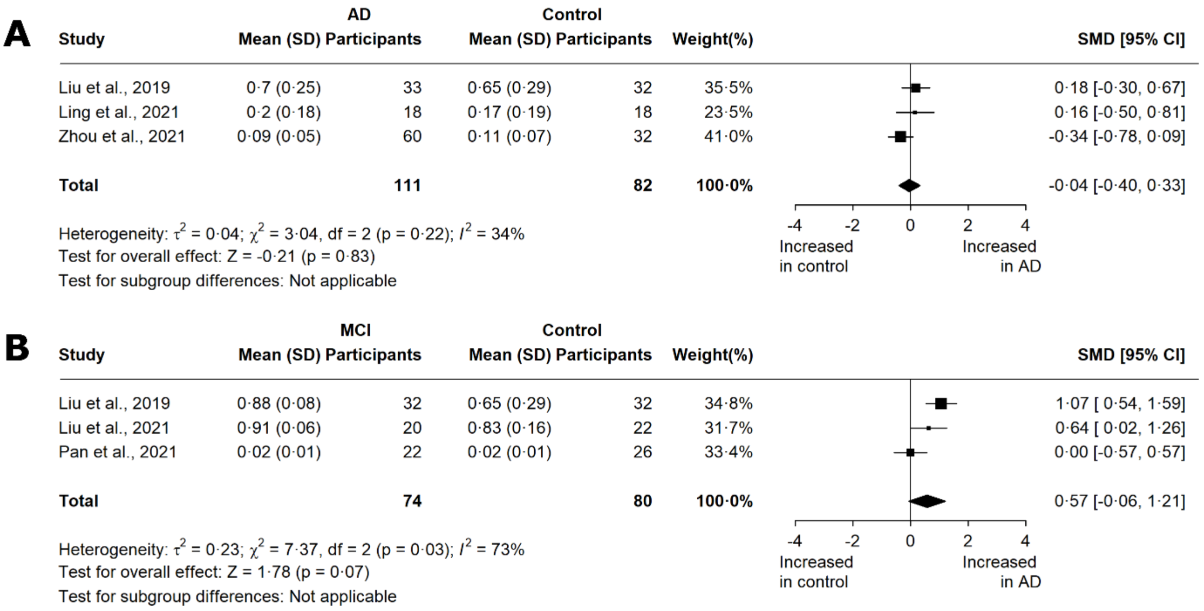

Fig 1. Changes in Simpson diversity index in gut microbiomes of AD and MCI patients. Simpson diversity in A) AD and B) MCI cohorts, grouped by study location. Data are mean (SD) and standard mean difference (95% CI) between groups by random-effect meta-analysis.

In terms of β-diversity indices, four studies have compared all three cohorts (AD, MCI and CN). Two studies reported obvious and significant differences in terms of Bray-Curtis and Jaccard distances[5,6]. Ueda et al.[7] reported no significant differences, which may be attributed to small sample sizes. Li and colleagues[14] reported that while there were differences among the groups, the difference between AD and MCI were not significant in terms of weighted UniFrac. Clear, significant differences between AD and CN have been reported in seven studies.[1,9-13] Two studies observed significant differences in terms of Bray-Curtis, but not UniFrac distance.[2,14] Three studies reported significant differences between MCI and CN groups.[1,15,16] Liu et al.[2] showed significant differences in terms of Bray-Curtis and weighted UniFrac but not unweighted UniFrac distances. Nagpal et al.[17] reported no significant differences, which may also be attributed to small sample sizes.

Members of *Bifidobacteriaceae* metabolise glucose to yield acetate and lactate.[18] Our meta-analysis showed a moderate but insignificant increase (SMD 0·70, 95% CI -0·93 to 2·33; p=0·40; figure S3) and considerable heterogeneity (I^2 = 98%; p<0·01). In contrast, *Bifidobacteriaceae* showed higher abundance in Parkinson's disease cohorts.[19]

Study	AD Mean (SD)	AD Participants	Control Mean (SD)	Control Participants	Weight(%)	SMD [95% CI]
Vogt et al., 2017	0·01 (0·02)	25	0·02 (0·03)	25	24·8%	-0·39 [-0·95, 0·17]
Li et al., 2019	0·03 (0·05)	30	0 (0·01)	30	24·9%	0·82 [0·29, 1·35]
Hou et al., 2021	0·01 (0·03)	30	0·05 (0·1)	47	25·1%	-0·49 [-0·96, -0·03]
Ling et al., 2021	0·16 (0·05)	100	0·05 (0·01)	71	25·2%	2·82 [2·40, 3·25]
**Total (95% CI)**		**185**		**173**	**100·0%**	0·70 [-0·93, 2·33]

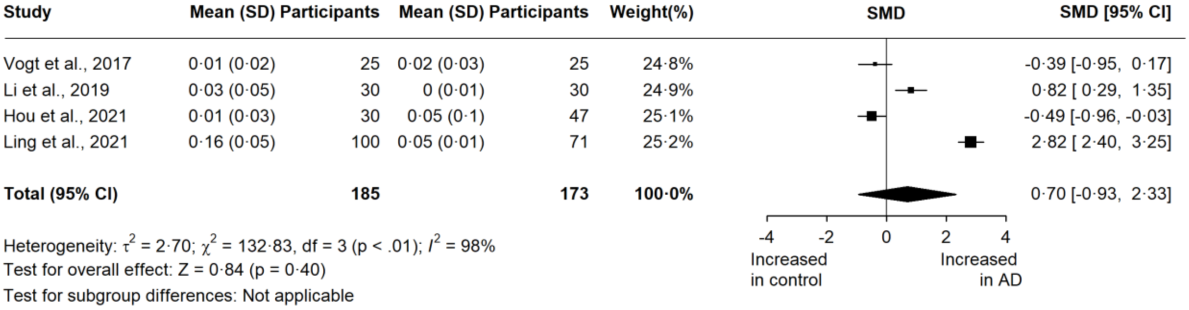

Heterogeneity: $\tau^2 = 2{\cdot}70$; $\chi^2 = 132{\cdot}83$, df = 3 ($p < .01$); $I^2 = 98\%$
Test for overall effect: Z = 0·84 (p = 0·40)
Test for subgroup differences: Not applicable

Fig 2. Relative abundance of family *Bifidobacteriaceae* in AD patients. Relative abundance of family *Bifidobacteriaceae* in AD cohorts. Data are mean (SD) and standard mean difference (95% CI) by random-effect meta-analysis.

Members of the *Blautia* genus are strictly anaerobic and non-motile. They utilize sugars (such as glucose and lactose) to produce acetate, succinate, lactate, and long-chain fatty acids. *Blautia* abundance has been negatively correlated with visceral fat accumulation and was reduced in patients with Type 2 diabetes, Crohn's disease and colorectal cancer,[20] which highlights its role in the maintaining gut health and alleviating inflammation. We observed practically no change in AD patients (SMD 0·08, 95% CI -0·49 to 0·65; p=0·78; figure S4) with substantial heterogeneity (I^2 = 80%; p<0·01). Thus, our analysis was inconclusive about the relationship between *Blautia* abundance and Alzheimer's disease.

Study	AD Mean (SD)	AD Participants	Control Mean (SD)	Control Participants	Weight(%)	SMD [95% CI]
Vogt et al., 2017	0·15 (0·07)	25	0·11 (0·07)	25	23·9%	0·56 [-0·00, 1·13]
Li et al., 2019	0·02 (0·02)	30	0·01 (0·01)	30	24·8%	0·62 [0·11, 1·14]
Liu et al., 2019	0·09 (0·08)	33	0·14 (0·12)	32	25·3%	-0·49 [-0·98, 0·01]
Hou et al., 2021	0·03 (0·03)	30	0·04 (0·03)	47	26·0%	-0·33 [-0·79, 0·13]
**Total**		**118**		**134**	**100·0%**	0·08 [-0·49, 0·65]

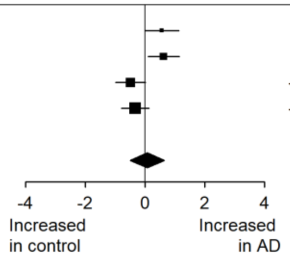

Heterogeneity: $\tau^2 = 0{\cdot}27$; $\chi^2 = 15{\cdot}00$, df = 3 ($p < .01$); $I^2 = 80\%$
Test for overall effect: Z = 0·28 (p = 0·78)
Test for subgroup differences: Not applicable

Fig 3. Relative abundance of genus *Blautia* in AD patients. Relative abundance of genus *Blautia* in AD cohorts. Data are mean (SD) and standard mean difference (95% CI) by random-effect meta-analysis.

References

1. Ling Z, Zhu M, Yan X et al. Structural and functional dysbiosis of fecal microbiota in Chinese patients with Alzheimer's disease. Front Cell Dev Biol 2021; 8: 634069. doi: 10.3389/fcell.2020.634069
2. Liu P, Wu L, Peng G et al. Altered microbiomes distinguish Alzheimer's disease from amnestic mild cognitive impairment and health in a Chinese cohort. Brain Behav Immun 2019; 80: 633–643. doi: 10.1016/j.bbi.2019.05.008
3. Zhou Y, Wang Y, Quan M, Zhao H, Jia J. Gut Microbiota Changes and Their Correlation with Cognitive and Neuropsychiatric Symptoms in Alzheimer's Disease. J Alzheimers Dis 2021; 81: 583–595. doi: 10.3233/JAD-201497
4. Liu P, Jia XZ, Chen Y et al. Gut microbiota interacts with intrinsic brain activity of patients with amnestic mild cognitive impairment. CNS Neurosci Ther 2021; 27: 163–173. doi: 10.1111/cns.13451
5. Duan M, Liu F, Fu H, Lu S, Wang T. Preoperative microbiomes and intestinal barrier function can differentiate prodromal Alzheimer's disease from normal neurocognition in elderly patients scheduled to undergo orthopedic surgery. Front Cell Infect Microbiol 2021; 11: 592842. doi: 10.3389/fcimb.2021.592842
6. Yıldırım S, Nalbantoğlu ÖU, Bayraktar A et al. Stratification of the Gut Microbiota Composition Landscape across the Alzheimer's Disease Continuum in a Turkish Cohort. mSystems 2022; 7: e0000422. doi: 10.1128/msystems.00004-22
7. Ueda A, Shinkai S, Shiroma H et al. Identification of Faecalibacterium prausnitzii strains for gut microbiome-based intervention in Alzheimer's-type dementia. Cell Rep Med 2021; 2: 100398. doi: 10.1016/j.xcrm.2021.100398
8. Li B, He Y, Ma J et al. Mild cognitive impairment has similar alterations as Alzheimer's disease in gut microbiota. Alzheimers Dement 2019; 15: 1357–1366. doi: 10.1016/j.jalz.2019.07.002
9. Pan Q, Li YQ, Guo K et al. Elderly Patients with Mild Cognitive Impairment Exhibit Altered Gut Microbiota Profiles. J Immunol Res 2021; 2021: 5578958. doi: 10.1155/2021/5578958
10. Guo M, Peng J, Huang X, Xiao L, Huang F, Zuo Z. Gut Microbiome Features of Chinese Patients Newly Diagnosed with Alzheimer's Disease or Mild Cognitive Impairment. J Alzheimers Dis 2021; 80: 299–310. doi: 10.3233/JAD-201040
11. Haran JP, Bhattarai SK, Foley SE et al. Alzheimer's Disease Microbiome Is Associated with Dysregulation of the Anti-Inflammatory P-Glycoprotein Pathway. mBio 2019; 10: e00632–19. doi: 10.1128/mBio.00632-19
12. Vogt NM, Kerby RL, Dill-McFarland KA et al. Gut microbiome alterations in Alzheimer's disease. Sci Rep 2017; 7: 13537. doi: 10.1038/s41598-017-13601-y
13. Xi J, Ding D, Zhu H et al. Disturbed microbial ecology in Alzheimer's disease: evidence from the gut microbiota and fecal metabolome. BMC Microbiol 2021; 21: 226. doi: 10.1186/s12866-021-02286-z
14. Zhuang ZQ, Shen LL, Li WW et al. Gut Microbiota is Altered in Patients with Alzheimer's Disease. J Alzheimers Dis 2018; 63: 1337–1346. doi: 10.3233/JAD-180176
15. Hou M, Xu G, Ran M, Luo W, Wang H. APOE-ε4 Carrier Status and Gut Microbiota Dysbiosis in Patients With Alzheimer Disease. Front Neurosci 2021; 15: 619051. doi: 10.3389/fnins.2021.619051
16. Khine WWT, Voong ML, Ng TKS et al. Mental awareness improved mild cognitive impairment and modulated gut microbiome. Aging 2020; 12: 24371–24393. doi: 10.18632/aging.202277
17. Nagpal R, Neth BJ, Wang S, Mishra SP, Craft S, Yadav H. Gut mycobiome and its interaction with diet, gut bacteria and Alzheimer's disease markers in subjects with mild cognitive impairment: A pilot study. EBioMedicine 2020; 59: 102950. doi: 10.1016/j.ebiom.2020.102950

18. Mattarelli P and Sgorbati B. Chemotaxonomic features in the Bifidobacteriaceae family, in Mattarelli P et al. (eds). The Bifidobacteria and Related Organisms. Academic Press, Cambridge, USA: 2018. pp 99–114.
19. Shen T, Yue Y, He T et al. The Association Between the Gut Microbiota and Parkinson's Disease, a Meta-Analysis. Front Aging Neurosci 2021; 13: 636545. doi: 10.3389/fnagi.2021.636545
20. Liu X, Mao B, Gu J et al. Blautia-a new functional genus with potential probiotic properties? Gut Microbes 2021; 13: 1–21. doi: 10.1080/19490976.2021.1875796
